# Addiction-Associated Genetic Variants Implicate Brain Cell Type- and Region-Specific Cis-Regulatory Elements in Addiction Neurobiology

**DOI:** 10.1523/JNEUROSCI.2534-20.2021

**Published:** 2021-10-27

**Authors:** Chaitanya Srinivasan, BaDoi N. Phan, Alyssa J. Lawler, Easwaran Ramamurthy, Michael Kleyman, Ashley R. Brown, Irene M. Kaplow, Morgan E. Wirthlin, Andreas R. Pfenning

**Affiliations:** ^1^Computational Biology Department, School of Computer Science, Carnegie Mellon University, Pittsburgh, Pennsylvania 15213; ^2^Medical Scientist Training Program, School of Medicine, University of Pittsburgh, Pittsburgh, Pennsylvania 15213; ^3^Department of Biological Sciences, Mellon College of Science, Carnegie Mellon University, Pittsburgh, Pennsylvania 15213; ^4^Neuroscience Institute, Carnegie Mellon University, Pittsburgh, Pennsylvania 15213

**Keywords:** addiction, deep learning, epigenetics, genomics, machine learning, neural circuits

## Abstract

Recent large genome-wide association studies have identified multiple confident risk loci linked to addiction-associated behavioral traits. Most genetic variants linked to addiction-associated traits lie in noncoding regions of the genome, likely disrupting *cis*-regulatory element (CRE) function. CREs tend to be highly cell type-specific and may contribute to the functional development of the neural circuits underlying addiction. Yet, a systematic approach for predicting the impact of risk variants on the CREs of specific cell populations is lacking. To dissect the cell types and brain regions underlying addiction-associated traits, we applied stratified linkage disequilibrium score regression to compare genome-wide association studies to genomic regions collected from human and mouse assays for open chromatin, which is associated with CRE activity. We found enrichment of addiction-associated variants in putative CREs marked by open chromatin in neuronal (NeuN^+^) nuclei collected from multiple prefrontal cortical areas and striatal regions known to play major roles in reward and addiction. To further dissect the cell type-specific basis of addiction-associated traits, we also identified enrichments in human orthologs of open chromatin regions of female and male mouse neuronal subtypes: cortical excitatory, D1, D2, and PV. Last, we developed machine learning models to predict mouse cell type-specific open chromatin, enabling us to further categorize human NeuN^+^ open chromatin regions into cortical excitatory or striatal D1 and D2 neurons and predict the functional impact of addiction-associated genetic variants. Our results suggest that different neuronal subtypes within the reward system play distinct roles in the variety of traits that contribute to addiction.

**SIGNIFICANCE STATEMENT** We combine statistical genetic and machine learning techniques to find that the predisposition to for nicotine, alcohol, and cannabis use behaviors can be partially explained by genetic variants in conserved regulatory elements within specific brain regions and neuronal subtypes of the reward system. Our computational framework can flexibly integrate open chromatin data across species to screen for putative causal variants in a cell type- and tissue-specific manner for numerous complex traits.

## Introduction

Substance use disorders have increased in prevalence over the last three decades, with an estimated 100 million cases worldwide (GBD 2016 Alcohol and Drug Use Collaborators, 2018; Eddie et al., 2019). Pharmacological interventions are limited in their ability to cure addiction because of physiological and logistic barriers (Pullen and Oser, 2014; Pear et al., 2019). As the societal epidemic of substance use grows, there is a greater need to understand the neurobiology of substance use behaviors and addiction.

The reward circuits co-opted in addiction as well as the associated neural cell types are highly conserved across primates and rodents (Monaco et al., 2015; Grillner and Robertson, 2016; Scaplen and Kaun, 2016; Hodge et al., 2019). Many studies have shown that addictive substances promote impulsive and compulsive behavior by activating the mesolimbic dopamine system, in which dopaminergic inputs from the VTA project to medium spiny neurons (MSNs) of the NAc in the ventral striatum (Koob and Volkow, 2010). Glutamatergic inputs to the NAc from the amygdala, frontal cortex, and hippocampus (HIPP) contribute to behavioral motivation through the extrapyramidal motor system (Koob and Volkow, 2010). Subsequently, the NAc sends outputs to nuclei of the ventral pallidum, which are critical for processing and modulating substance reward signal (Koob and Volkow, 2010). The development of compulsive substance-seeking is hypothesized to be linked to recruitment of the dorsal striatum, which together with the prefrontal cortical regions regulates a variety of reward- and addiction-related phenotypes (Koob and Volkow, 2010; Goldstein and Volkow, 2011). These findings emphasize that substance abuse behavior involves the interplay of the brain regions and cell types that make up the reward system.

Increasing evidence reveals strong genetic links to substance use risk (Pasman et al., 2018; Karlsson Linnér et al., 2019; M. Liu et al., 2019) and substance use disorder (Kendler and Prescott, 1998a,b; Dick, 2016; Waaktaar et al., 2018; Erzurumluoglu et al., 2020). Genome-wide association studies (GWASs) report that genetic risk for substance use shares underlying architecture with other neuropsychiatric disorders (Pasman et al., 2018; M. Liu et al., 2019). These risk variants tend to lie in noncoding regions of the human genome rather than the protein-coding regions that have clearer functional consequence to link genes to traits (Jensen, 2016). These genetic variants, including single nucleotide polymorphisms (SNPs), can disrupt transcription factor binding in *cis*-regulatory elements (CREs) with varying impact on gene regulation and downstream neural circuitry. Many CREs have tissue- and cell type-specific activity (Roadmap Epigenomics Consortium et al., 2015), suggesting that cell types and tissues underlying addiction may be uniquely targeted by genetic variants at these CREs. GWASs for nicotine-, alcohol- (M. Liu et al., 2019), and cannabis-use traits (Pasman et al., 2018) have identified multiple confident risk loci and SNPs linked to addiction-associated phenotypes with brain specificity, yet their effects on the CREs of specific brain regions and cell types involved in addiction pathophysiology are an open area of inquiry.

Comparisons of GWASs to functional annotations of the human genome have yielded estimates that >90% of SNPs associated with complex phenotypes lie within potentially functional noncoding regions, which are marked by epigenetic features, including open chromatin (Maurano et al., 2012; Finucane et al., 2015). Linkage disequilibrium (LD) of significant SNPs complicates the identification of causal variants contributing to genetic risk (Bush and Moore, 2012), as multiple SNPs that are tightly linked will inherently all have similar association with a disorder, even if not all of them are necessarily causal. Regression of SNP LD scores against GWAS summary statistics (LDSC regression) is the dominant method for relating human genetics to functional annotations. LDSC regression partitions risk SNPs identified by GWAS into the tissues or cell types in which genetic variation in CREs may contribute to heritability of complex traits (Finucane et al., 2015; Visscher et al., 2017). Yet, the functional consequences of risk SNPs in CRE sequences cannot be reliably inferred from DNA sequences alone (Shlyueva et al., 2014). By synthesizing recent developments in epigenomic assays (Buenrostro et al., 2013; Mo et al., 2015; Tak and Farnham, 2015) and machine learning (Ghandi et al., 2014; Zhou and Troyanskaya, 2015; Kelley et al., 2016, 2018; D. Lee, 2016), it is possible to predict cell types affected by addiction-associated genetic variation and propose cell type-specific hypotheses on the pathogenesis of addiction.

Here, we implement a framework that links the genetic predisposition to addiction-associated traits to specific brain regions and cell types within them by identifying those that have open chromatin regions (OCRs) that are enriched for SNPs identified by GWASs. We first intersect SNPs measured by GWASs with human and mouse bulk tissue- and cell type-specific OCRs to identify putative region- and cell type-specific CREs that may be impacted by genetic variation associated with addiction-related traits. To overcome limits of cellular resolution in the human brain, we apply convolutional neural network (CNN) models trained on transgenically labeled neuron populations in the reward system of mice to predict the cell type specificity of GWAS-associated SNPs in the human genome. We further apply these models to the problem of screening for putative causal SNPs within dense loci reported in GWAS for addiction-associated traits. This pipeline, to our knowledge, describes the first integrative analyses across species, brain regions, and cell types to screen for candidate causal addiction-associated genetic risk variants in dense loci with numerous significant SNPs in LD.

## Materials and Methods

### ATAC-seq data processing pipeline

We processed raw FASTQ files of ATAC-seq experiments with the official ENCODE ATAC-seq pipeline accessed by https://github.com/ENCODE-DCC/atac-seq-pipeline. We ran this pipeline using the mm10 genome assembly for mouse and the hg38 genome for human with the following settings: smooth_win = 150, multimapping = 0, idr_thresh = 0.1, cap_num_peak = 300,000, keep_irregular_chr_in_bfilt_peak = true. We grouped biological replicates (e.g., samples from the same tissue region or condition) when processing data to obtain individual de-duplicated, filtered bam files. We removed samples that had low periodicity indicated by ENCODE quality control metrics (https://www.encodeproject.org/atac-seq/) and reprocessed the remaining replicates with the pipeline. Using the high-quality replicates, we obtained reproducible (IDR) peaks for each condition. Unless otherwise stated, we used the “optimal” reproducible set of peaks for downstream analyses.

### Publicly available datasets

#### NeuN-sorted ATAC-seq of human postmortem brain (Fullard et al., 2018)

We identified OCRs overlapping addiction-related variants through analysis of human postmortem brain ATAC-seq in which cells were sorted into NeuN^+^ and NeuN^–^ groups via fluorescence-activated nuclei sorting; the brain regions we used were dorsolateral PFC (DLPFC), orbitofrontal cortex (OFC), ventrolateral PFC (VLPFC), ACC, superior temporal gyrus (STC), inferior temporal gyrus (ITC), primary motor cortex (PMC), insula (INS), primary visual cortex (PVC), amygdala, HIPP, mediodorsal thalamus (MDT), NAc, and putamen (PUT). We downloaded data from the Sequence Read Archive through Gene Expression Omnibus (GEO) accession #GSE96949. We separated samples by cell type and reprocessed them with the ENCODE pipeline as detailed above, aligning reads to hg38. All datasets were high quality according to the ENCODE metrics for epigenomic datasets (https://www.encodeproject.org/atac-seq/). We used the “optimal reproducible peaks” for each cell type and brain region as foregrounds in GWAS LDSC enrichment with the Honeybadger2 OCR set as the background set (see LDSC regression GWAS enrichment backgrounds).

#### Single-cell chromatin accessibility profiling (Corces et al., 2020)

We downloaded 24 clusters of IDR peaks of human isocortex, striatum, HIPP, and substantia nigra in BED format through GEO accession #GSE147672. These clusters represent cell populations defined by Corces et al. (2020) from the measured brain regions. We assigned clusters to cell populations as described by Corces et al. (2020): astrocyte (AST) (clusters 13, 17), hippocampal excitatory (clusters 3, 4), isocortical AST (cluster 15), isocortical excitatory (cluster 1), isocortical inhibitory (cluster 11), microglia (cluster 24), neuron (cluster 7), nigral AST (cluster 14), nigral neurons (clusters 5, 6), nigral oligodendrocyte precursor (cluster 10), oligodendrocyte (clusters 19-23), oligodendrocyte precursor (clusters 8, 9), striatal AST (cluster 16), and striatal inhibitory cells (clusters 2, 12). We did not include cluster 18, which corresponds to a doublet. We merged coordinates from clusters assigned to the same cell types to define foreground sets for LDSC regression GWAS enrichment. We merged the foreground sets with the Honeybadger2 OCR set to define the background set (LDSC regression GWAS enrichment backgrounds).

#### Human occipital cortex scTHS-seq (Lake et al., 2018)

We downloaded BED-formatted cell type-specific differential OCRs from occipital cortex scTHS-seq of excitatory neurons (EXC), inhibitory neurons (INs), ASTs, endothelial cells, oligodendrocyte precursor cells, oligodendrocytes, and microglia (MIC) from the GEO subseries #GSE97887. We used the hg38 OCR coordinates as foregrounds in LDSC regression GWAS enrichment with the Honeybadger2 OCR set as the background set (LDSC regression GWAS Enrichment Backgrounds).

#### Mouse INTACT-sorted nuclei ATAC-seq (Mo et al., 2015)

We downloaded FASTQ files of *R26-CAG-LSL-Sun1-sfGFP-Myc* transgenic mouse lines for cell type-specific ATAC-seq performed using the INTACT method from the accession #GSE63137. Mo et al. (2015) isolated INTACT-enriched nuclei from three cell types: EXC (*Camk2a-cre*), vasoactive intestinal peptide neurons (VIP, *Vip-cre*), and parvalbumin neurons (PV, *Pvalb-cre*). We reprocessed the data with the Kundaje laboratory open chromatin pipeline using the mm10 genome (https://github.com/kundajelab/atac_dnase_pipelines). We mapped reproducible mouse ATAC-seq peaks for each cell type to hg38 using halLiftover with the 12-mammals Cactus alignment (Paten et al., 2011; Hickey et al., 2013) followed by HALPER (Zhang et al., 2020) (mapping mouse OCR orthologs) to produce a foreground set of orthologous human sequences for LDSC regression GWAS enrichment (Finucane et al., 2018). We mapped the ENCODE mm10 DNaseI-hypersensitive peak set (Yue et al., 2014) to hg38 (mapping mouse OCR orthologs) and used successfully mapped hg38 orthologs of mm10 OCRs as background set for mouse foreground enrichments. Furthermore, we used this dataset to evaluate differential accessibility in cre-dependent Sun1-GFP Nuclear Anchored Independent Labeled (cSNAIL)-INTACT PV and PV-negative ATAC-seq samples and develop CNN models of cell type-specific open chromatin (see methods below).

Human negative control foregrounds (ENCODE Project Consortium, 2012; Thurman et al., 2012; Davis et al., 2018; Cannon et al., 2019): We downloaded raw ATAC-seq profiles of human adult female and male stomach ATAC-seq (ENCSR337UIU, ENCSR851SBY, respectively), female human embryonic liver DNase-seq (ENCSR562FNN), and human embryonic lung DNase-seq (ENCSR582IPV) from https://www.encodeproject.org/. We processed these files using the ENCODE pipeline as detailed above to obtain optimal reproducible hg38 peaks. We also downloaded BED files of human adipocyte and preadipocyte ATAC-seq profiles generated by Cannon et al. (2019), from GEO accession number #GSE110734. We mapped these BED coordinates from hg19 to hg38 using liftOver to define negative control foregrounds for human LDSC regression GWAS enrichment. We merged the human negative control foregrounds and Fullard et al. (2018), foregrounds with the Honeybadger2 OCR set to define the background for human negative control foreground enrichments.

#### Human-orthologous negative control foregrounds (C. Liu et al., 2019)

We also downloaded raw ATAC-seq data profiled in female mouse kidney, female mouse liver, and male mouse lung generated by C. Liu et al. (2019), from Sequence Read Archive accession #SRP167062 to define human-orthologous negative control foregrounds. We processed these files using the ENCODE pipeline as detailed above to get optimal reproducible peaks. We mapped optimal reproducible peaks from mm10 to hg38 using halLiftover with the 12-mammals Cactus alignment followed by HALPER (mapping mouse OCR orthologs) to define negative control foregrounds for human-orthologous LDSC GWAS enrichments. We merged all human orthologous foregrounds with the human orthologs of the ENCODE mm10 DNaseI-hypersensitive peak set to define a background for human-orthologous LDSC GWAS enrichments.

### Mapping mouse OCR orthologs

We used halLiftover (Hickey et al., 2013) with the 12-mammals Cactus alignment (Paten et al., 2011) followed by HALPER (https://github.com/pfenninglab/halLiftover-postprocessing) (Zhang et al., 2020) to map mm10 mouse reproducible OCRs to hg38 human orthologs to perform LDSC regression GWAS enrichment. The Cactus multiple sequence alignment file (Paten et al., 2011) has 12 genomes, including mm10 and hg38, aligned in a reference-free manner, allowing us to leverage multispecies alignments to confidently identify orthologous regions across species. halLiftover uses a Cactus-format multiple species alignment to map BED coordinates from a query species to orthologous coordinates of a target species, and HALPER constructs contiguous orthologs from the outputs of halLiftover (Zhang et al., 2020). We ran the orthologFind.py function from HALPER on the outputs of halLiftover using the following parameters: -max_frac 5.0 -min_frac 0.05 -protect_dist 5 -narrowPeak -mult_keepone. In general, 70% of mouse brain ATAC-seq reproducible peaks were able to be mapped to confident human orthologs. To map the ENCODE mm10 mouse DHS background, which does not contain summit information, to hg38, we used the mouse coordinates of position with the most species aligned in a region to define the summit. Only for the mm10 mouse DHS background set, for which a significant proportion of regions could not be confidently mapped to hg38, we flanked the original assembly coordinates by 300 bp to increase OCR mapping from 54% to 64%.

### Experimental design

To augment and compare to mouse cell type-specific ATAC-seq datasets generated in this study, we performed bulk tissue ATAC-seq from cortex (CTX) and dorsal striatum/NAc (CPU) of 7- and 12-week-old C57Bl/6J mice (*N* = 2 each age) from both sexes (Extended Data Table 4-1) as described by Buenrostro et al. (2015) with the following minor differences in buffers and reagents. We killed mice with isoflurane, rapidly decapitated to extract the brain, and sectioned it in ice-cold oxygenated aCSF (119 mm NaCl, 2.5 mm KCl, 1 mm NaH_2_PO_4_ (monobasic), 26.2 mm NaHCO_3_, 11 mm glucose) at 200 μm sections on a vibratome (Leica Microsystems, VT1200). We further micro-dissected sections for cortex and dorsal striatum on a stereo microscope and transferred dissected regions into chilled lysis buffer (Buenrostro et al., 2015). We Dounce homogenized the dissected brains in 5 ml of lysis buffer with the loose pestle (Pestle A) in a 15 ml glass Dounce homogenizer (Pyrex, #7722-15). We washed nuclei lysate off the pestle with 5 ml of lysis buffer and filtered the nuclei through a 70 μm cell strainer into a 50 ml conical tube. We washed the Dounce homogenizer again with 10 ml of BL buffer and transferred the lysate through the 70 μm filter (Foxx, 1170C02). We pelleted the 20 ml of nuclei lysate at 2000 × *g* for 10 min in a refrigerated centrifuge at 4°C. We discarded the supernatant and resuspended the nuclei in 100-300 μl of water to approximate a concentration of 1-2 million nuclei/ml. We filtered the nuclei suspension through a 40 μm cell strainer. We stained a sample of nuclei with DAPI (Invitrogen, #D1206) and counted the sample to measure 50k nuclei per ATAC-seq transposition reaction. The remaining steps follow the Buenrostro et al. (2015) protocol for tagmentation and library amplification. We shallowly sequenced barcoded ATAC-seq libraries at 1-5 million reads per sample on an Illumina MiSeq and processed individual samples through the ENCODE pipeline for initial quality control. We used these QC measures (clear periodicity, library complexity, and minimal bottlenecking) to filter out low-quality samples and repooled a balanced library for paired-end deep sequencing on an Illumina NextSeq to target 30 million uniquely mapped fragments per sample after mitochondrial DNA and PCR duplicate removal. These raw sequencing files entered processing through the ENCODE ATAC-seq pipeline as above by merging technical replicates and grouping biological replicates by brain region for each pipeline run.

10.1523/JNEUROSCI.2534-20.2021.t4-1 Extended Data Table 4-1 cSNAIL sample description: genotype, number of replicates, sex, region and number of replicates per region, and cell type information for cSNAIL samples. Download Table 4-1, DOCX file.

The cSNAIL genome (pAAV-Ef1a-DIO-Sun1-Gfp-WPRE-pA) contains *loxP* sites to invert the *Sun1-Gfp* fusion gene and integrate into the nuclear membrane of cells expressing the *Cre* gene, allowing these cell populations to be profiled for various genomic assays (Lawler et al., 2020). We packaged the cSNAIL genome with AAV variant PHP.eB (pUCmini-iCAP-PHP.eB) in AAVpro(R) 293T cells (Takara, catalog #632273). Viviana Gradinaru provided us with the pUCmini-iCAP-PHP.eB (http://n2t.net/addgene:103005; RRID: Addgene_103005) (Chan et al., 2017). We precipitated viral particles with polyethylene glycol, isolated with ultracentrifugation on an iodixanol density gradient, and purified in PBS with centrifugation washes and 0.2 μm syringe filtration. We injected each mouse with 4.0 × 10^11^vg into the retro-orbital cavity under isoflurane anesthesia. We allowed the virus to incubate in the animal for 3-4 weeks to reach peak expression. We closely monitored the health of the animals throughout the length of the virus incubation and did not note any concerns.

On the day of the cSNAIL mouse or bulk tissue ATAC-seq experiments, we dissected brain regions from fresh tissue and extracted nuclei in the same manner as described for bulk tissue experiments. Then, we sorted the nuclei suspension into Sun1GFP^+^ (Cre^+^) and Sun1GFP^–^ (Cre^–^) fractions using affinity purification with Protein G Dynabeads (Thermo Fisher Scientific, catalog 10004D). A preclearing incubation with beads and nuclei for 10-15 min removes effects from nonspecific binding events. Next, we incubated the remaining free nuclei with anti-GFP antibody (Invitrogen, #G10362) for 30 min to bind Sun1GFP. Finally, we added new beads to the solution to conjugate with the antibody and incubated the reaction for an additional 20 min. The preclear step and all incubations took place in wash buffer (0.25 m sucrose, 25 mm KCl, 5 mm MgCl_2_, 20 mm Tricine with KOH to pH 7.8, and 0.4% IGEPAL) at 4°C with end-to-end rotation. After the binding process, we separated bead-bound nuclei on a magnet, washed 3 times with wash buffer, and filtered through a 20 μm filter to ensure purity. We resuspended nuclei in nuclease-free water for input into the ATAC-seq tagmentation reaction. We performed nuclei quantification and tagmentation in the same manner described for bulk tissue ATAC-seq above.

We list in Extended Data Table 4-1 the number of animals, the genotypes, and which regions collected for ATAC-seq experiments in this study. All transgenic mouse strains in this study were originally generated on C57BL/6J backgrounds, and lines were maintained on a C57BL/6J background throughout breeding. The general breeding strategy was homozygous transgenic mice with C57BL/6J mice to produce heterozygous transgenic offspring for experiments, except for Sst-Cre mice, which were homozygous for the transgene. To minimize genetic drift within an isolated population, breeding C57BL/6J mice and transgenic mice were routinely refreshed after 1-5 generations with stock animals from The Jackson Laboratory or from other colonies at Carnegie Mellon University. The mice did not contain additional transgenes outside of cell type-specific Cre. *N* = 2 *PValb-2a-Cre* samples from CPU/NAc region had received a sham surgery with saline injection into the external globus pallidus 5 d before they were killed. *N* = 2 *D1-Cre* samples from both CPU and NAc regions had received headcap surgeries 3 weeks before they were killed. Both *PValb-2a-Cre* and *D1-Cre* were overall healthy at time of death. We collected cSNAIL samples from both sexes where possible (Extended Data Table 4-1).

### Statistical analyses

We created a consensus set of nonoverlapping IDR peaks from the ATAC-seq pipeline for cSNAIL ATAC-seq and Mo et al. (2015), INTACT samples (Tissue: Ctx, CPU, and NAc; Celltype: EXC, PV, SST, VIP, D1, D2). We extended the peak set 200 bp upstream and downstream, count overlapping fragments with Rsubread version 2.0.1 using the de-duplicated BAM files from the pipeline (Liao et al., 2014), and created with DESeq2 version 1.26.0 a variance-stabilized count matrix aware of experimental Group (combination of Tissue and Celltype) with ∼Group (Love et al., 2014). We plotted the principal component analysis in Figure 4*A* for the first two components with this variance-stabilized count matrix. We used Deeptools version 3.5.0 to convert the same BAM files to normalized bigWig files and average over replicates of the same Group (Ramírez et al., 2016). We plotted the tracks using pyGenomeTracks version 3.5 around marker genes for each cell type (*Slc17a7*, *Drd1*, *Adora2a*, *Pvalb*, *Sst*, *Vip*; see Fig. 4*B*). (Ramírez et al., 2018). We computed the mean accessibility for each group 2 kb upstream and downstream the transcription start sites (TSSs) and correlated log_10_ (TSS accessibility + 1) with gene expression log_10_(meta gene counts + 1) of Drop-Seq annotated cell types from PFC and striatum (Saunders et al., 2018). We used the Saunders et al. (2018) tissue subcluster metagene profiles (sum of gene expression in all cells) and summed subclusters to cluster-level metagene profiles. Most tissue cluster metagene profiles corresponded to cSNAIL ATAC-seq celltype and tissue profiles, with the exception of cSNAIL cortical PV^+^ samples were matched to Saunders et al. (2018) cortical MGE^+^ interneuron clusters.

We computed the conditioned heritability of CREs for GWAS variants using the stratified LDSC regression pipeline for cell type-specific enrichment as outlined in https://github.com/bulik/ldsc/wiki/Cell type-specific-analyses (Bulik-Sullivan et al., 2015b). We downloaded the GWAS summary statistics files and processed them with the LDSC munge_sumstats function to filter rare or poorly imputed SNPs with default parameters. We munged the summary statistics files for HapMap3 SNPs, excluding the MHC regions downloaded at http://ldsc.broadinstitute.org/static/media/w_hm3.noMHC.snplist.zip. We inspected GWAS file to ensure the effect allele, non-effect allele, sample size, *p* value, and signed summary statistic for each SNP in each GWAS were included and appropriate for LDSC. The addiction-associated GWASs measure genetic predisposition for age of smoking initiation (AgeofInitiation) (M. Liu et al., 2019), heaviness of smoking (CigarettesPerDay) (M. Liu et al., 2019), having ever regularly smoked (SmokingInitiation) (M. Liu et al., 2019), current versus former smokers (SmokingCessation) (M. Liu et al., 2019), alcoholic drinks per week (DrinksPerWeek) (M. Liu et al., 2019), cannabis consumption (Cannabis) (Pasman et al., 2018), and risk tolerance (RiskyBehavior) (Karlsson Linnér et al., 2019). GWAS traits related to addiction include multisite chronic pain (ChronicPain) (Johnston et al., 2019) and number of coffee cups drank per day (CoffeePerDay) (Coffee and Caffeine Genetics Consortium et al., 2015). Other addiction-related traits come from underpowered GWAS, including opioid dependence (OpioidDep) (Cheng et al., 2018), cocaine dependence (CocaineDep) (Cabana-Domínguez et al., 2019), and diagnosis of obsessive-compulsive disorder (OCD) (International Obsessive Compulsive Disorder Foundation Genetics Collaborative and OCD Collaborative Genetics Association Studies, 2018). GWASs from strong brain-related traits used are schizophrenia risk (Schizophrenia) (Schizophrenia Working Group of the Psychiatric Genomics Consortium, 2014), highest level of educational attainment (EduAttain) (J. J. Lee et al., 2018), and sleep duration (SleepDuration) (Dashti et al., 2019). The non–brain-related traits measure genetic liability for lean body mass (LBM) (Zillikens et al., 2017), bone mineral density (BMD) (Kemp et al., 2017), and coronary artery disease (CAD) (Howson et al., 2017).

We estimated LD scores for each foreground set and corresponding background set (see LDSC Regression GWAS Enrichment Backgrounds) with the LDSC regression pipeline make_annot and ldsc functions using hg38 1000 Genomes European Phase 3 European super-population (1000G EUR) cohort plink files downloaded from https://data.broadinstitute.org/alkesgroup/LDSCORE/GRCh38/. An example of an ATAC-seq optimal set of reproducible peaks mapped to hg38 in narrowPeak format is annotated with 1000G EUR LD scores using the following call:

python make_annot.py\
–bed-file optimal_peak.narrowPeak.gz\
–bimfile 1000G.EUR.hg38.${chr}.bim\
–annot-file foreground.${chr}.annot

We downloaded the baseline v1.2 files for cell type-specific enrichment in hg38 coordinates from the same link above as well as the corresponding weights weights.hm3_noMHC file, excluding the MHC region from https://data.broadinstitute.org/alkesgroup/LDSCORE/. HapMap SNPs and corresponding weights file used in the LDSC analyses only refer to the SNP rsIDs, rather than genomic coordinates, so only the baseline and LD statistics used to annotate the foreground and background files must be in hg38 coordinates. In accordance with the LDSC regression script input format, we created an enrichment.ldcts file listing the annotated foreground/background pair for each foreground set. We estimated the conditional heritability enrichment using the ldsc function, which integrates the foreground and background LD score estimates, munged GWAS SNP data, baseline variant data, and variants weights. The final function call to GWAS enrichment was as follows:

python ldsc.py –h2-cts $Munged_GWAS\
–ref-ld-chr baseline_v1.2/baseline.\
–w-ld-chr weights.hm3_noMHC.\
–ref-ld-chr-cts enrichment.ldcts\
–out $Output_Label

**Figure 1.**
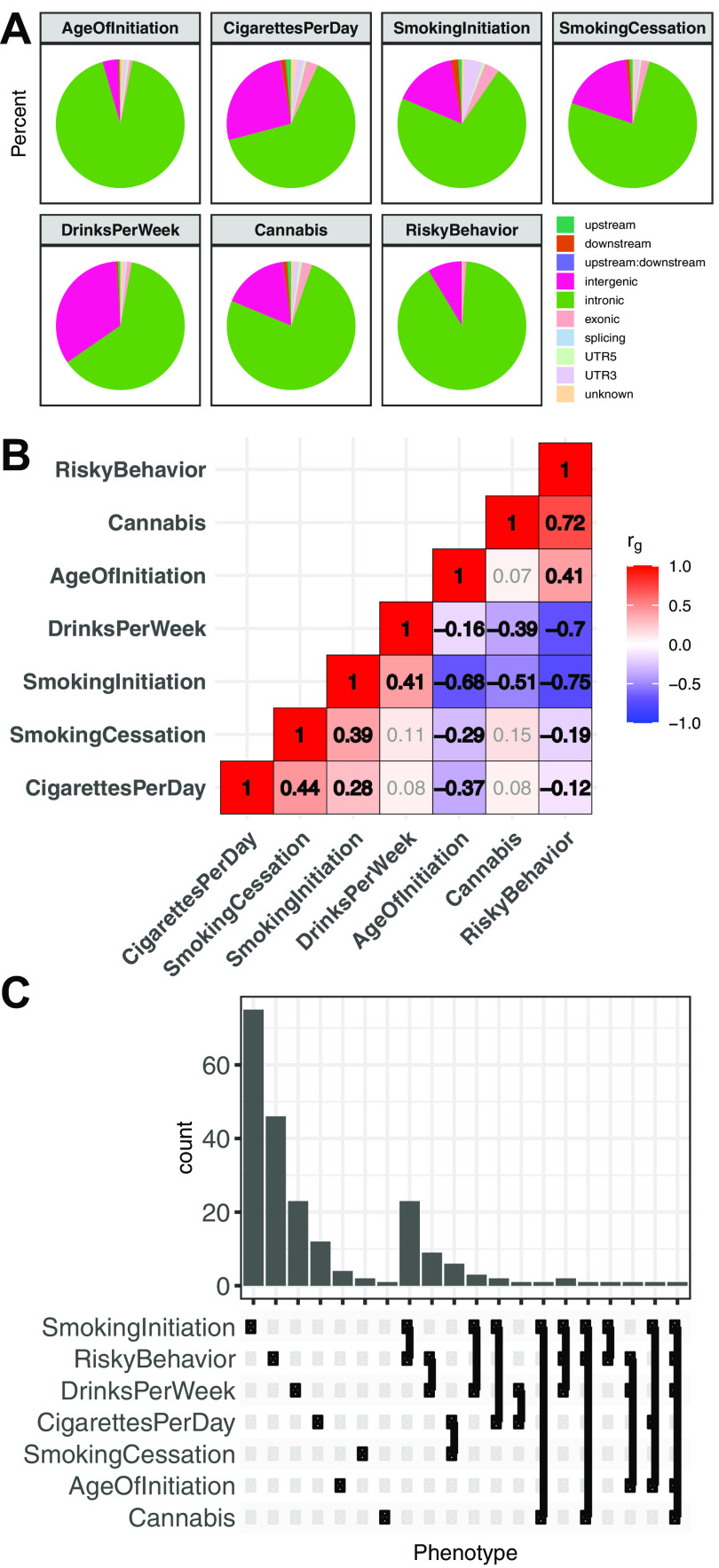
Shared and unique genetic architecture of genetic risk variants of addiction-associated traits. ***A***, Pie chart of ANNOVAR-annotated (K. Wang et al., 2010) SNP function of addiction-associated trait lead and off-lead SNPs in LD *R*^2^ > 0.8. Dark colors represent untranscribed/noncoding annotations; light colors represent transcribed/exonic annotations. SNP annotation labels are according to ANNOVAR using ENSEMBL build 85 gene annotations (see Materials and Methods). ***B***, Pairwise LDSC genetic correlation (r_g_) matrix of seven addiction-associated traits. Bold represents FDR-significant correlations. Gray represents nonsignificant correlations (FDR < 0.05). ***C***, UpSet plot of nonoverlapping genomic loci shared or unique to each addiction-associated trait. Genomic loci are clustered and identified by shared GWAS-significant SNPs and genomic region overlap.

The pipeline was run using the –h2-cts parameter produces the conditional coefficient estimate of tau_C (the additive difference in heritability per SNP in SNPs inside versus outside the foreground conditional on the background and baseline annotations) (Finucane et al., 2015, 2018), coefficient error, and coefficient *p* value estimates. We adjusted for multiple testing using the false discovery rate (FDR) on coefficient *p* values of the LD score regression coefficients (α = 0.05) on all 18 GWAS traits intersected on within the same foreground/background set. A significant FDR value indicates enrichment of the foreground genomic regions for GWAS SNPs relative to the background. Last, we computed genetic correlations in Figure 1*A* between GWAS of addiction-associated traits using the pre-munged summary statistics as described by Bulik-Sullivan et al. (2015a).

We trained a set of CNN models to learn the regulatory code of a given cell type from the DNA sequences underlying the cell type's OCRs. The models take in one-hot encoded 501 bp genomic sequences to predict 1 for an OCR or 0 for a non-OCR sequence. Positive sequences were centered on IDR peak summits that are annotated to be in introns and nonpromoter noncoding regions, and negative sequences are ∼10 times the number of positives sequences that are G/C-matched and not overlapping IDR peaks. We excluded promoters, which we defined as within 5000 bp of the TSS (based on GRCH38.p12), and exons because distal sequences have been shown to confer more enhancer cell type specificity and be more predictive of expression levels of regulated genes (Roadmap Epigenomics Consortium et al., 2015). We constructed the negative set by first building a sequence repository $BGDIR according to https://bitbucket.org/CBGR/biasaway_background_construction/src/master/ from the mouse mm10 genome using 501 bp sequences. Then we used the biasaway (Worsley Hunt et al., 2014; Khan et al., 2020) command-line interface to generate negative sequences with the matching nucleotide distribution along a sliding window of the 501 bp IDR peak sequence as follows:

biasaway c –foreground $FORGEGROUND_FASTA –nfold 10 –deviation 2.6 –step 50 –seed 1 –winlen 100 –bgdirectory $BGDIR

We used a fivefold cross-validation chromosome hold-out scheme to train 5 models per set of IDR peaks, enabling us to evaluate the stability and consistency of learned regulatory patterns. (A model that was training a fold did not see sequences during training from the validation set for that fold, and no model saw the test set until final model performance evaluation.) Sequences from these chromosomes were used as the validation set for each fold as follows:

fold1: {chr6, chr13, chr21}
fold2: {chr7, chr14, chr18}
fold3: {chr11, chr17, chrX}
fold4: {chr9, chr12}
fold5: {chr10, chr8}.

We used sequences from chromosomes {chr1, ch2, chr19} for the test set.

We trained the models with Keras version 2.3.0-tf (https://keras.io/) implemented through Tensorflow version 2.2.0 and used stochastic gradient descent with Nesterov momentum to learn model parameters that minimized the binary cross entropy loss. All models used the same CNN architecture after a grid-search of hyperparameters found stable and high validation performance by area under the precision-recall curve (auPRC) in an architecture with five Conv1D layers [kernel_size = 11, filters = 200, activation = relu, kernel_regularizer = l2(1e-10)] with a Dropout layer (rate = 0.25) between each pair of consecutive layers, then one MaxPooling1D layer (pool_size = 26, strides = 26), one Flatten layer, one Dense layer [units = 300, activation = relu, kernel_regularizer = l2(1e-10)], one Dropout layer (rate = 0.25), a final output Dense layer [units = 1, activation = sigmoid, kernel_regularizer = l2(1e-10)], and a final Dropout layer (rate = 0.25) before the sigmoid output layer to train each fold with batch_size = 1000, epochs = 23, num_cycles = 2.35, base_learning_rate = 1e-2, max_learning_rate = 1e-1, base_momentum = 0.85, max_momentum = 0.99. With these hyperparameters, we trained models across folds to predict positive OCRs of all measured cell types against an ∼1:10 positive:negative class ratio. We computed classifier performance metrics, including weighted accuracy (using threshold = 0.5), weighted f1_score (using threshold = 0.5), area under receiver operating characteristic, and auPRC. We selected the reported hyperparameters because they maximized the validation auPRC, which we prioritized because of the class imbalance. We report the test set auPRC, F1 score, and false positive rate in Figure 7*A*. We provide both the scripts and trained Keras models at https://github.com/pfenninglab/addiction_gwas_enrichment.

**Figure 2.**
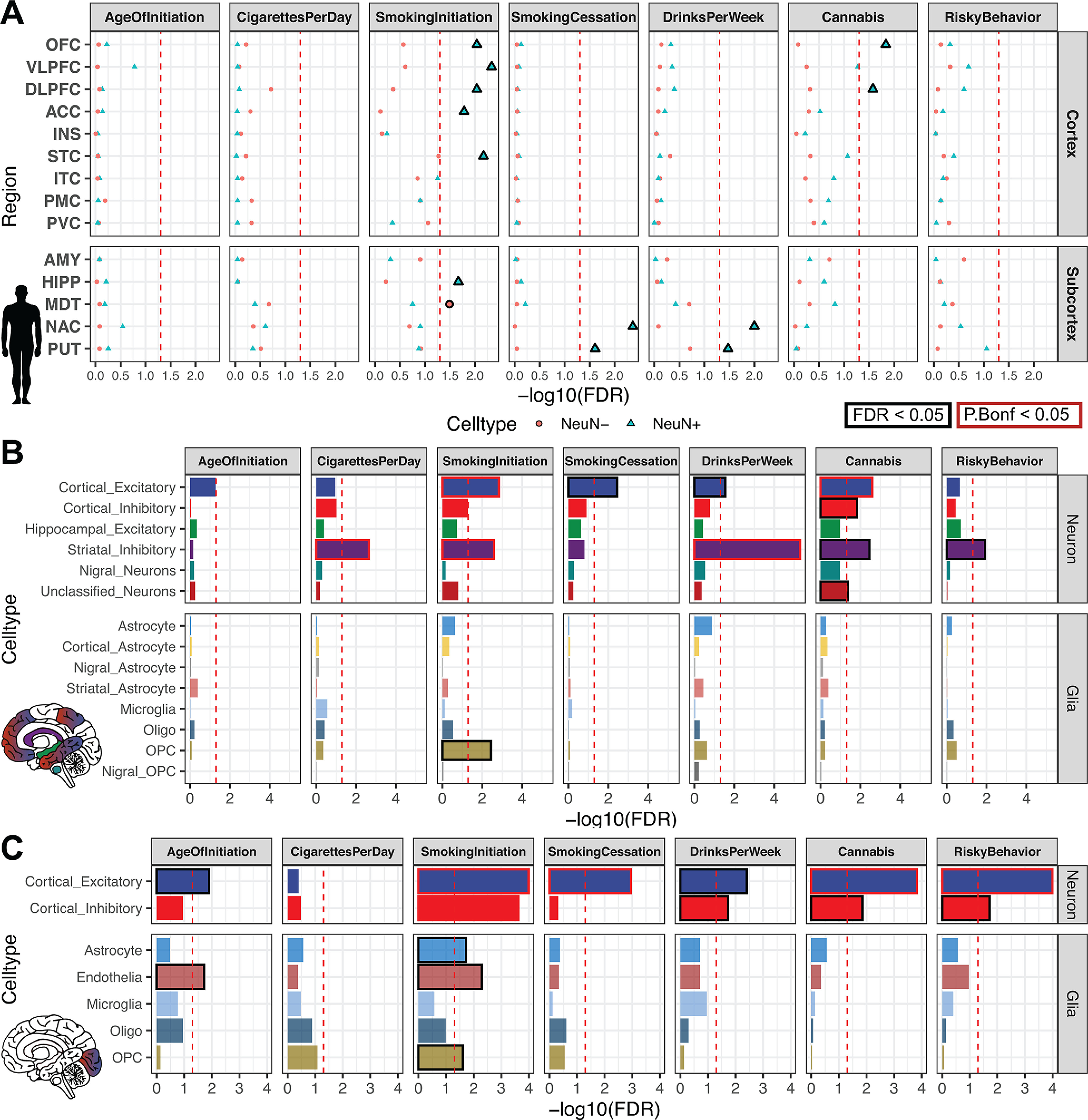
Substance use and risky behavior GWAS risk variants enrich within reward region- and cell type-specific epigenomic profiles. Stratified LDSC regression (GWAS enrichment) finds enrichment of substance use and risky behavior traits in region-specific and cell type-specific open chromatin profiles of human postmortem brain. ***A***, GWAS enrichment FDRs in ATAC-seq of 14 postmortem human brain regions coupled with NeuN-labeled fluorescence-activated nuclei sorting (Fullard et al., 2018). Brain regions are stratified by cortical and subcortical regions, with cortical regions ordered frontal to caudal. Sorted cell types within each brain region are denoted as follows: blue triangle represents NeuN^+^/neuronal; red circle represents NeuN^–^/glial. FDR adjustment was performed across all enrichments on the Fullard et al. (2018), dataset for Figure 2. Brain regions reported to be significantly enriched (FDR ≤ 0.05, black; Bonferroni *p* value ≤ 0.05, red) are plotted with bolded points. Dashed red line indicates the significance threshold. ***B***, Barplot of GWAS enrichment FDRs in single-cell open chromatin profiles of cell clusters in isocortex, HIPP, and striatum (Corces et al., 2020). Cell types in brain regions that are significantly enriched (FDR ≤ 0.05) are plotted with bolded bars. Dashed red line indicates the significance threshold. ***C***, Barplot of GWAS enrichment FDRs in single-cell THS-seq OCRs of major cell clusters in occipital cortex (Lake et al., 2018). Cell types in brain regions that are significantly enriched (FDR ≤ 0.05) are plotted with bolded bars. Dashed red line indicates the significance threshold. Traits assessed are age of smoking initiation (AgeofInitiation), average number of cigarettes per day for ever smokers (CigarettesPerDay), having ever regularly smoked (SmokingInitiation), current versus former smokers (SmokingCessation), number of alcoholic drinks per week (DrinksPerWeek) (C. Liu et al., 2019), lifetime cannabis use (Cannabis) (Pasman et al., 2018), and risky behavior (RiskyBehavior) (Karlsson Linnér et al., 2019). AMY, Amygdala; Ast, AST; End, endothelial; Ex, EXC; In, IN; Mic, microglia; Oli, oligodendrocyte; Opc, oligodendrocyte precursor.

We used CNN model scores to classify whether a peak from Fullard et al. (2018), NeuN^+^ open chromatin data are active in a neuronal subtype (EXC, D1, D2). We took NeuN^+^ IDR “optimal peaks” from regions significantly enriched for addiction-associated traits (OFC, VLPFC, DLPFC, ACC, STC, PUT, NAc; see Fig. 2*A*), extracted 501 bp DNA sequences of each centered on the summit, and scored each peak with cell type-specific machine learning models trained with the appropriate tissue context (e.g., score cortical NeuN^+^ peaks with a model trained with cortical EXC cell type). We averaged scores across models from different cross-validation folds from the same cell types and classified NeuN^+^ peaks with scores >0.5 as active in that cell type, as this threshold was the most discriminative in classifying positive validation set sequences (see Fig. 7*B*). We defined these CNN-prioritized peaks as foregrounds for the LDSC regression GWAS enrichment analyses as described above. We created a consensus set of peaks merging all model-prioritized peaks and the Honeybadger2 set of OCRs to be the matched background, and we performed GWAS enrichment and computed FDR for all 18 GWAS traits (only enrichments for addiction-associated GWAS shown; see Fig. 8).

We collected the addiction-associated SNPs by submitting the summary statistics files for the seven addiction-associated traits {AgeofInitiation (M. Liu et al., 2019), CigarettesPerDay (M. Liu et al., 2019), SmokingInitiation (M. Liu et al., 2019), SmokingCessation (M. Liu et al., 2019), DrinksPerWeek (M. Liu et al., 2019), Cannabis (Pasman et al., 2018), RiskyBehavior (Karlsson Linnér et al., 2019)} to the FUMA webserver (Watanabe et al., 2017). FUMA computed LD R^2^ based on the 1000 Genomes European (1000G EUR) super-population reference (1000 Genomes Project Consortium et al., 2015) via PLINK (Purcell et al., 2007), linked GWAS-significant lead SNPs to off-lead SNPs in LD with the lead, and annotated functional consequences of genetic variants via ANNOVAR based on ENSEMBL build 85 human gene annotations (K. Wang et al., 2010). ANNOVAR functional gene annotations for a SNP are as defined in the primary publication and online: https://annovar.openbioinformatics.org/en/latest/user-guide/gene/. We scored all effect and non-effect alleles with each set of CNN models, averaged predictions across folds, and calibrated CNN scores that predict activity using the set of validation positive OCRs. We computed the ΔSNP probability effect by taking the difference between the effect allele and the non-effect allele. Most SNPs reported by GWAS are not expected to be the causal variant for a trait, so the distribution of ΔSNP probability can be used to define a null distribution. We computed the *p* value that an allele has a non-zero ΔSNP probability by fitting a normal distribution of null ΔSNP probabilities. We corrected for multiple testing using the method swfdr version 1.12.0 to compute *q* values to control for a FDR conditioned on potentially informative covariates (Boca and Leek, 2018). Weighted FDR-correction methods, including swfdr, have been shown to be robust to uninformative covariates and increase power to detect real differences for informative covariates while controlling false discoveries (Korthauer et al., 2019). We conditioned the proportion of expected null *p* values on the following covariates (see Fig. 7*E*, Step 4): the difference in GC content of the 501 surrounding the SNP compared with the average GC content of positive sequences used to train each model (GC content), the minor allele frequency based on the European ancestry subjects in the 1000G reference panel, whether the SNP overlapped a Fullard et al. (2018), NeuN^+^ OCR (inNeuN peak), and whether an SNP was fine-mapped and predicted to be causal by CAUSALdb using the European LD structure and an ensemble of statistical fine-mapping tools (isCausal) (FINEMAP, CAVIARBF, PAINTOR) (W. Chen et al., 2015; Benner et al., 2016; Kichaev et al., 2017; J. Wang et al., 2020). We applied an α of 0.05 on the false-discovery *q* values for all 14,790 SNPs scored across 5 sets of CNNs to determine significantly large enough ΔSNP effects.

To accompany cell type-specific activity predictions, we downloaded SNPs that are reported as *cis* expression quantitative trait loci (eQTL) in human cortex, frontal cortex (DLPFC), ACC, caudate, PUT, and the NAc from the GTEX Consortium from https://www.gtexportal.org/home/datasets (GTEx Consortium, 2013, 2015). We identified genes for which at least one of the 170 SNPs is an eQTL and plotted them as arcs in Figures 6 and 9*B*. Locus plots are generated with pyGenomeTracks version 3.5 tools (Ramírez et al., 2018).

For Figure 9*A*, we compared calibrated SNP probabilities of the either effect or non-effect allele across each model and grouped them by whether they overlapped a cortical or striatal NeuN^+^ OCR, NeuN^–^ OCR, both, or neither, depending on whether the model was for EXC or D1/D2 neuronal subtypes, respectively. We computed two-tailed *t* tests between groups and corrected for multiple comparisons with the familywise Bonferroni method for *N* = 18 tests from three models and (4 choose 2) six possible comparisons per model. * *p* < 0.05/*N*. ** *p* < 0.01/*N*. *** *p* < 0.001/*N*.

### LDSC regression GWAS enrichment backgrounds

We found that LDSC regression GWAS enrichment analysis is sensitive to the selected background set of matched regions. To construct appropriate background sets for each GWAS enrichment, we used the ENCODE and RoadMap Honeybadger2 (Roadmap Epigenomics Consortium et al., 2015) and Mouse DHS peak sets for the respective human and mouse-based open chromatin GWAS enrichment. The Honeybadger2 set, downloaded from https://personal.broadinstitute.org/meuleman/reg2map/, consists of DNaseI-hypersensitive OCRs across 53 epigenomes consisting of promoter, enhancer, and dyadic regions. Honeybadger2 is an appropriate epigenetic reference for enrichment of cell type-specific open chromatin from various foregrounds, such as Fullard et al. (2018) and Lake et al. (2018). Honeybadger2 regions allow the LDSC algorithm to properly account for the heritability from OCRs of a particular cell type or regions rather than because they tend to be more conserved, are enriched for ubiquitously active transcription factor motifs, or other factors distinguishing open chromatin from heterochromatin. The human orthologs of the ENCODE Mouse DHS peak set, downloaded through the ENCODE ATAC-seq pipeline at https://storage.googleapis.com/encode-pipeline-genome-data/mm10/ataqc/mm10_univ_dhs_ucsc.bed.gz, is a set of peaks combined from mouse DNaseI-hypersensitivity OCRs from ENCODE and provides a background for human orthologs of mouse OCRs. The mm10 mouse DHS regions were mapped to hg38 as described in Mapping mouse OCR orthologs. For each respective foreground-background pairing, the foreground regions were merged with the background reference to ensure the background always contained the foreground set. The mouse background was used to calculate the significance of the relationship between mouse OCRs and GWAS for addiction-associated traits to control for a possible association between the degree to which a region is conserved and its likelihood in influencing the predisposition to an addiction-associated trait.

### Interpretation of CNN models

To ensure that the classification task decisions relied on biologically relevant sequence signatures and not artifacts, we performed model interpretation using Deep SHAP version 0.37.0 (Štrumbelj and Kononenko, 2014; Shrikumar et al., 2017) and TF-MoDISco (Shrikumar et al., 2018). For a random subsample of 2000 true positive sequences from the validation set, we generated per-base importance scores and hypothetical importance scores relative to a reference set of 500 true negative sequences from the validation set. These scores describe the contribution of each base toward a positive model classification, which is a predicted OCR in the given cell type. TF-MoDISco is an importance score-aware method that clusters commonly important subsequences, called “seqlets,” to construct the motifs that the model learned. We ran TF-MoDISco version 0.4.2.3 with the options sliding_window_size = 11, flank_size = 3, min_seqlets_per_task = 3000, trim_to_window_size = 11, initial_flank_to_add = 3, final_flank_to_add = 3, kmer_len = 7, num_gaps = 1, and num_mismatches = 1. The resulting motifs were filtered to remove rare patterns with <100 supporting seqlets. Then, the motifs were visualized and associated with known motifs using Tomtom (Gupta et al., 2007) version 5.3.3 with the HOCOMOCO v11 FULL database and default parameters (Extended Data Fig. 7-1).

### Data availability

Code used to run intermediate and final analyses reported in this paper are available on the GitHub page: https://github.com/pfenninglab/addiction_gwas_enrichment. Sequencing output files for data generated in this study are deposited on the GEO at accession GSE161374.

## Results

### Genetic risk for substance use traits is associated with the neuronal epigenomes of reward areas

Recent well-powered GWASs have identified dozens of candidate genetic risk loci associated with seven addiction-associated traits: age of smoking initiation (AgeOfInitiation), average number of cigarettes smoked per day (CigarettesPerDay), having ever regularly smoked (SmokingInitiation), being a former versus current smoker (SmokingCessation), the number of alcoholic drinks per week (DrinksPerWeek), lifetime cannabis use (Cannabis), and risk tolerance (RiskyBehavior) (Pasman et al., 2018; Karlsson Linnér et al., 2019; M. Liu et al., 2019). These GWASs measure reward, risk tolerance, and various substance use behaviors, thereby providing a means of studying genetic variation associated with addiction. We found that 72%-98% of addiction-associated genetic variants lie in noncoding regions of the genome (Fig. 1*A*). Of those risk variants, 47%-85% lie in introns, which is a substantial overrepresentation in each GWAS (odds ratio, OR_AgeOfInitiation_ = 2.3, OR_Cannabis_ = 2.3, OR_CigarettesPerDay_ = 1.4, OR_DrinksPerWeek_ = 1.6, OR_RiskyBehavior_ = 1.4, OR_SmokingCessation_ = 1.8, OR_SmokingInitiation_ =1.3, Fisher's Exact P_Bonferroni_ < 8 × 10^−79^). Furthermore, pairwise genetic correlations of risk alleles in these seven GWASs indicated shared and distinct genetic architecture across addiction-associated traits (r_g_; Fig. 1*B*). Although common genetic variants are shared between addiction-associated traits on a genome-wide scale, the reported significant loci are often unique to a particular trait and are densely packed with SNPs in high LD (Fig. 1*C*). SNPs that are associated with the seven traits span 205 nonoverlapping loci across the human genome and include on average 71 SNPs (minimum 1, median 22.5, maximum 1780) within each locus that are either genome-wide significant (P_GWAS_ < 5 × 10^−8^) or in high LD with the lead SNPs (*R*^2^ > 0.8; Extended Data Fig. 7-1).

We investigated whether genetic variants implicated by addiction-associated GWASs show a tendency to cluster at putative CREs of the brain using a stratified LDSC regression approach (see Statistical analyses), which looks for an enrichment of significant SNPs from GWAS in human annotations (Bulik-Sullivan et al., 2015b; Finucane et al., 2018). We applied LDSC to compare the seven addiction-associated GWASs to OCR annotations of sorted neuronal (NeuN^+^) and glial (NeuN^–^) nuclei across 14 brain regions (Fullard et al., 2018) (Fig. 2*A*). We found that genetic variants associated with SmokingInitiation, SmokingCessation, DrinksPerWeek, and Cannabis significantly enriched in NeuN^+^ OCRs of brain regions known and speculated to contribute to reward and addiction (Volkow and Morales, 2015) (FDR < 0.05). We found that genetic variants associated with SmokingInitiation and Cannabis shared enrichment in NeuN^+^ prefrontal cortical OCRs (from OFC and DLPFC; SmokingInitiation-OFC FDR = 9.1E-03, Cannabis-OFC FDR = 1.4E-02, SmokingInitiation-DLPFC FDR = 9.1E-03, Cannabis_DLFC FDR = 2.6E-02), whereas those associated with SmokingCessation and DrinksPerWeek shared enrichment in NeuN^+^ striatal OCRs (both PUT and NAc; SmokingCessation-PUT FDR = 2.5E-02, SmokingCessation-NAC FDR = 4.3E-03, DrinksPerWeek-PUT FDR = 3.3E-02, DrinksPerWeek-NAC FDR = 1.0E-02). The enrichments of NeuN^+^ OCRs indicate that genetic variation in epigenomes of neuronal populations from frontal cortex and striatum contribute to addiction liability. The difference in NeuN^+^ enrichments between regions across addiction-associated traits can likely be explained by the difference in proportions and identities of neuronal subtypes of each area, so we sought to dissect the different neuronal subtypes contributing to these enrichments.

Broad marker-gene based labeling approaches, such as using NeuN to label neurons, do not capture the rich diversity of neuronal subtypes; bulk NeuN^+^ open chromatin signal represents an average signal from heterogeneous neuronal subtypes, each with distinct epigenomic landscapes, gene regulation, and network connectivity. Hence, NeuN-labeled open chromatin profiles likely do not capture OCRs unique to less populous neuronal subtypes. The difference in proportions of neuronal subtypes between brain regions may also contribute to brain region-specific NeuN^+^ OCR enrichment for GWAS variants of addiction-associated traits. We therefore applied LDSC regression GWAS enrichment on single-cell open chromatin profiles from human postmortem isocortical, striatal, hippocampal, nigral (Fig. 2*B*) and occipital cortical cell types (Fig. 2*C*) (Lake et al., 2018; Corces et al., 2020). We found that addiction-associated genetic variants largely enriched in both excitatory and inhibitory neuronal OCRs. Genetic variants associated with SmokingInitiation (FDR = 1.4E-03, P_Bonferroni_ = 1.8E-02), SmokingCessation (FDR = 3.6E-03), DrinksPerWeek (FDR = 3.6E-03), and Cannabis enriched in isocortical EXC OCRs (Fig. 2*B*). We found enrichment of genetic variants associated with CigarettesPerDay (FDR = 2.2E-03, P_Bonferroni_ = 3.3E-02), SmokingInitiation (FDR = 2.5E-03, P_Bonferroni_ = 4.4E-02) DrinksPerWeek (FDR = 4.8E-06, P_Bonferroni_ = 3.4E-05), Cannabis (FDR = 3.4E-03), and RiskyBehavior (FDR = 1.1E-02) in striatal INs. Genetic variants associated with Cannabis also enriched in isocortical IN (FDR = 1.5E-02) and unclassified neuron OCRs (FDR = 4.4E-02). Among the glial cell types, only oligodendrocyte precursor cell OCRs were enriched for an addiction-associated trait (SmokingInitiation; FDR = 3.6E-03). We found enrichment of genetic variants associated with AgeOfInitiation (FDR = 1.2E-02) and SmokingCessation (FDR = 1.0E-04, P_Bonferroni_ = 6.1E-04) in OCRs of occipital cortical EXCs. We found no enrichment of genetic variants associated with CigarettesPerDay for OCRs of occipital cortex cell types. Genetic variants associated with SmokingInitiation, which enriched in AST (FDR = 1.8E-02), endothelial (FDR = 5.1E-03), inhibitory (FDR = 2.4E-04, P_Bonferroni_ = 2.1E-03), and oligodendrocyte precursor cell OCRs (FDR = 2.5E-02) from occipital cortex, shared enrichment in NeuN^–^ OCRs of MDT (Fig. 2*A*; FDR = 3.2E-02). Interestingly, genetic variants associated with SmokingCessation, which showed enrichment for striatal NeuN^+^ OCRs, enriched only for OCRs of occipital cortical EXCs and not cortical INs (FDR = 1.1E-03, P_Bonferroni_ = 1.3E-02). Sorted bulk ATAC-seq only showed enrichment of Smoking Cessation-associated genetic variants in OCRs of NeuN^+^ striatal regions, which are largely composed of inhibitory MSNs. We overall found that the enrichments of addiction-associated genetic variants in Corces et al. (2020), isocortex OCRs agreed with those in Lake et al. (2018), occipital cortex OCRs. Single-cell epigenomics of human postmortem brain can further dissect the genetic risk for substance-use traits into neuronal subtypes that otherwise would not be parsed with bulk tissue assays.

**Figure 3.**
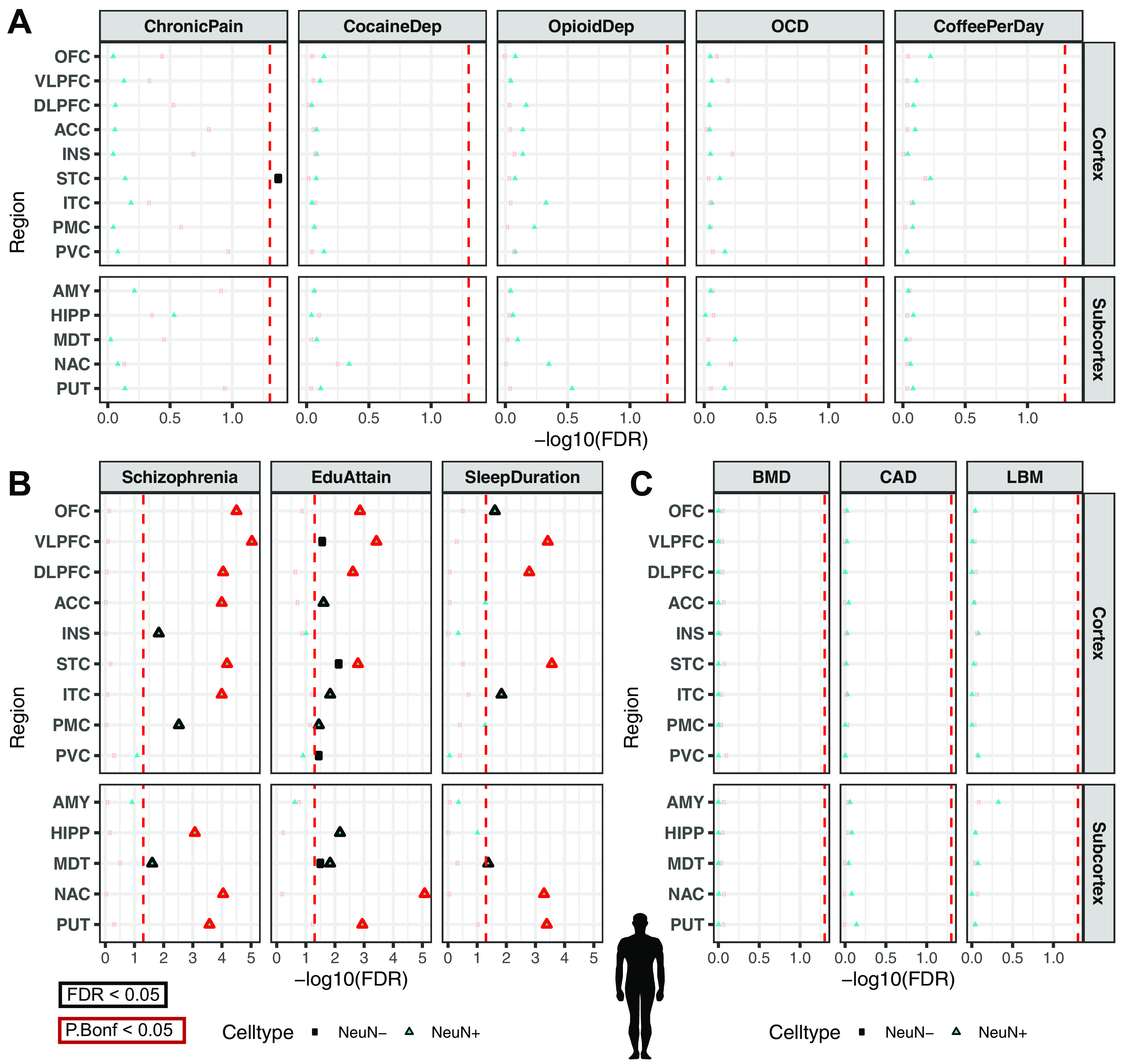
Sensitivity of stratified LDSC regression for cell type- and region-specific in the GWAS trait enrichment requires well-powered GWAS in relevant cell types. GWAS enrichment plots with FDRs in ATAC-seq of 14 postmortem human brain regions coupled with NeuN-labeled fluorescence-activated nuclei sorting (Fullard et al., 2018). Regions are stratified by cortical and subcortical regions, with cortical regions ordered frontal to caudal. Sorted cell types within each brain region are denoted by shape as follows: blue triangle represents NeuN^+^/neuronal; red circle represents NeuN^–^/glial. Cell types in brain regions that are enriched at FDR ≤ 0.05 are plotted with bigger shapes and with black outlines and enriched at Bonferroni *p* value ≤ 0.05 with red outlines. ***A***, GWAS enrichment of addiction- or substance use-associated traits: multisite chronic pain (ChronicPain) (Johnston et al., 2019), cocaine dependence (CocaineDep) (Cabana-Domínguez et al., 2019), opioid dependence (OpioidDep) (Cheng et al., 2018), diagnosis of OCD (International Obsessive Compulsive Disorder Foundation Genetics Collaborative and OCD Collaborative Genetics Association Studies, 2018), and cups of coffee drank per day (CoffeePerDay) (Coffee and Caffeine Genetics Consortium et al., 2015). The GWASs for OCD, opioid dependence, and cocaine dependence are reportedly underpowered to detect genetic liability for these traits (*N*_case_ < 5000). ***B***, GWAS enrichment in well-powered brain-related traits showss cell type- and region-specific enrichment: educational attainment (EduAttain) (J. J. Lee et al., 2018), schizophrenia risk (Schizophrenia) (Schizophrenia Working Group of the Psychiatric Genomics Consortium, 2014), and habitual sleep duration (SleepDuration) (Dashti et al., 2019). ***C***, GWAS enrichment in non–brain-associated traits does not show cell type- or region-specific enrichment: heel BMD (Kemp et al., 2017), CAD (Howson et al., 2017), and LBM (Zillikens et al., 2017).

We confirmed that our pipeline for LDSC regression on NeuN-sorted OCRs from 14 brain regions is able to reproduce the GWAS enrichments published by Fullard et al. (2018). While our approach uses OCRs from reproducible ATAC-seq peaks rather than differentially accessible peaks, we found consistent enrichments of genetic variants associated with schizophrenia risk (Schizophrenia; VLPFC NeuN^+^ FDR = 9.3E-06, OFC NeuN^+^ FDR = 3.1E-05, STC NeuN^+^ FDR = 6.6E-05, NAC NeuN^+^ FDR = 9.1E-05, DLPFC NeuN^+^ FDR = 9.1E-05, ACC NeuN^+^ FDR = 1.0E-04, ITC NeuN^+^ FDR = 1.0E-04, PUT NeuN^+^ FDR = 2.6E-04, HIPP NeuN^+^ FDR = 8.4E-04, PMC NeuN^+^ FDR = 2.9E-03, INS NeuN^+^ FDR = 1.4E-02, MDT NeuN^+^ FDR = 2.4E-02), highest level of educational attainment (EduAttain; NAC NeuN^+^ FDR = 8.4E-06, VLPFC NeuN^+^ FDR = 3.7E-04, PUT NeuN^+^ FDR = 1.1E-03, OFC NeuN^+^ FDR = 1.3E-03, STC NeuN^+^ FDR = 1.6E-03, DLPFC NeuN^+^ FDR = 2.4E-03, STC NeuN^–^ FDR = 6.7E-03, HIPP NeuN^+^ FDR = 6.7E-03, ITC NeuN^+^ FDR = 1.4E-02, MDT NeuN^+^ FDR = 1.4E-02, VLPFC NeuN^–^ FDR = 2.4E-02, ACC NeuN^+^ FDR = 2.4E-02, MDT NeuN^–^ FDR = 2.8E-02, PVC NeuN^–^ FDR = 3.2E-02, PMC NeuN^+^ FDR = 3.5E-02), and habitual sleep duration (SleepDuration; STC NeuN^+^ FDR = 2.7E-04, VLPFC NeuN^+^ FDR = 3.7E-04, PUT NeuN^+^ FDR = 4.0E-04, NAC NeuN^+^ FDR = 5.0E-04, DLPFC NeuN^+^ FDR = 1.6E-03, ITC NeuN^+^ FDR = 1.4E-02, OFC NeuN^+^ FDR = 2.4E-03, MDT NeuN^+^ FDR = 4.1E-03) (Fig. 3*B*). We did not find enrichment in brain OCRs of genetic variants identified in several low-powered GWAS [cocaine dependence (CocaineDep) (Cabana-Domínguez et al., 2019), opioid dependence (OpioidDep) (Cheng et al., 2018), and OCD (International Obsessive Compulsive Disorder Foundation Genetics Collaborative and OCD Collaborative Genetics Association Studies, 2018)], each of which had included <5000 individuals with the trait (Fig. 3*A*). In addition, we found no enrichments in brain OCR for several well-powered studies of traits related to addiction behaviors, including multisite chronic pain (ChronicPain) (Johnston et al., 2019) and quantity in cups of coffee drank per day (CoffeePerDay) (Coffee and Caffeine Genetics Consortium et al., 2015). We also found no enrichment in brain OCRs for anthropometric traits, including CAD (Howson et al., 2017), BMD (Kemp et al., 2017), and LBM (Zillikens et al., 2017) (Fig. 3*B*,*C*). Last, we validated that human OCRs from nonbrain tissues would not enrich for risk variants associated with brain traits. We gathered publicly available OCRs from stomach ATAC-seq, adipocyte ATAC-seq, preadipocyte ATAC-seq, liver DNase-seq, and lung DNase-seq profiles (ENCODE Project Consortium, 2012; Thurman et al., 2012; Davis et al., 2018; Cannon et al., 2019) (see Fig. 6*D*) and performed LDSC regression on the total 18 GWAS from above. To our expectation, we did not find enrichments of stomach, liver, or lung OCRs for genetic variants associated with brain-related traits. We did find enrichment of BMD in lung OCRs (FDR = 9.1E-04, P_Bonferroni_ = 9.1E-04), a connection previously recognized (I. S. Lee et al., 2016; Kim et al., 2019; Zeng et al., 2019). The secondary GWAS enrichments in other traits and foregrounds demonstrate two trends: a GWAS trait would enrich if the GWAS was properly powered to detect genetic risk variants, and the foreground regions are from cell types or tissue of that trait's potential etiologic origin.

### Mouse-human conserved cell type-specific open chromatin enrich for addiction risk loci

**Figure 4.**
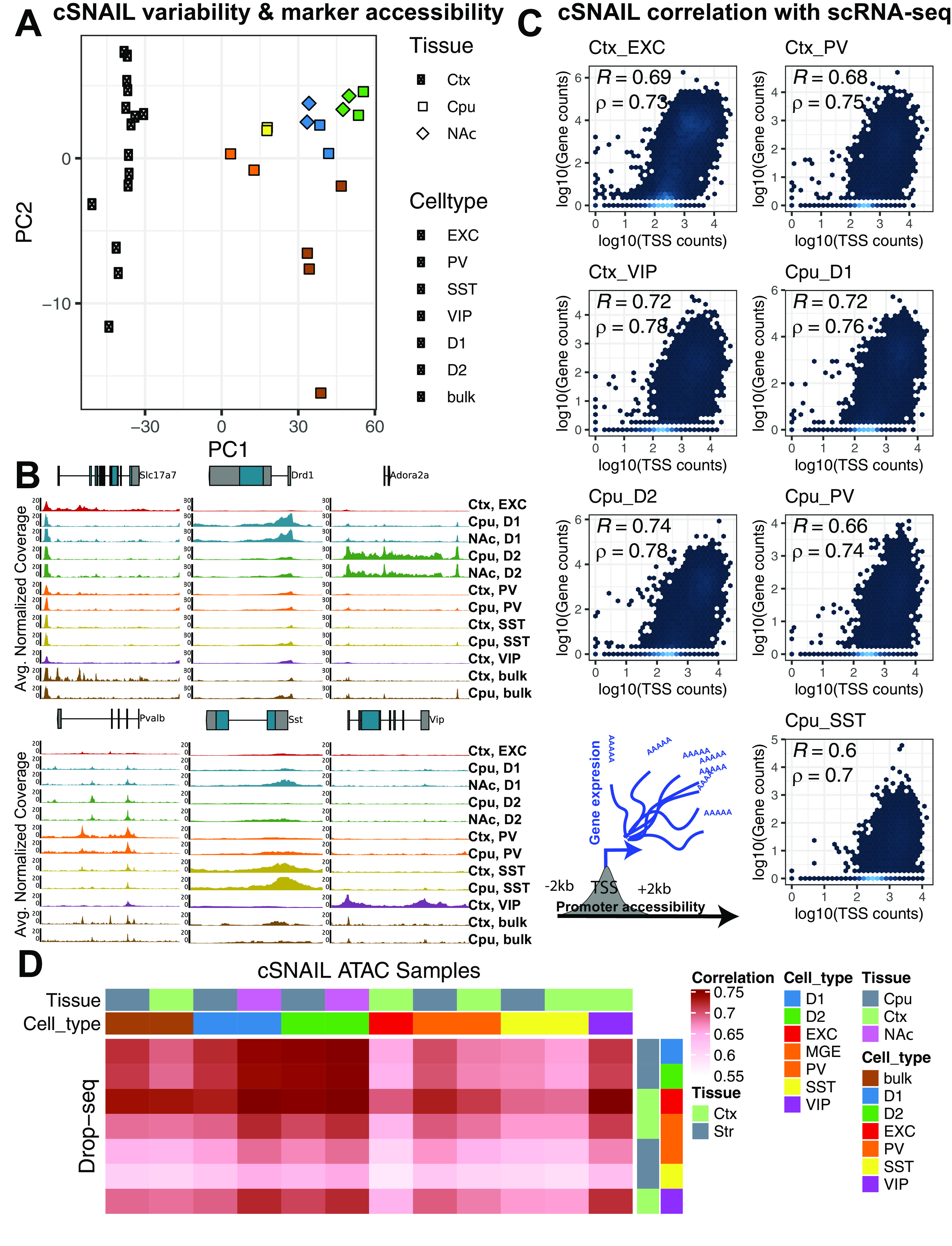
Cell type specificity of cSNAIL ATAC-seq in mouse cortex and striatum. ***A***, Principal component plots of chromatin accessibility counts from cSNAIL ATAC-seq from *cre*-driver lines (see Materials and Methods; sample sizes in Extended Data Table 4-1). Major axes of variation separate cell types by tissue source (PC1) and cell type versus bulk ATAC-seq (PC2). ***B***, Normalized coverage track plots around marker genes demarcating cell type specificity of cSNAIL ATAC-seq samples. ***C***, Density correlation plot of normalized chromatin accessibility log counts around the TSSs correlated with matched pseudo-bulk cell type log gene counts from Drop-seq of mouse cortex and striatum (Saunders et al., 2018). Drop-seq cell types meta-gene profiles report sum gene counts for cell clusters from frontal cortex and striatum. *R* and ρ indicate Pearson's and Spearman's correlation, respectively. ***D***, Pairwise correlation matrix of TSS chromatin accessibility log counts with Drop-seq pseudo-bulk log gene counts from cortical and striatal cell clusters.

**Figure 5.**
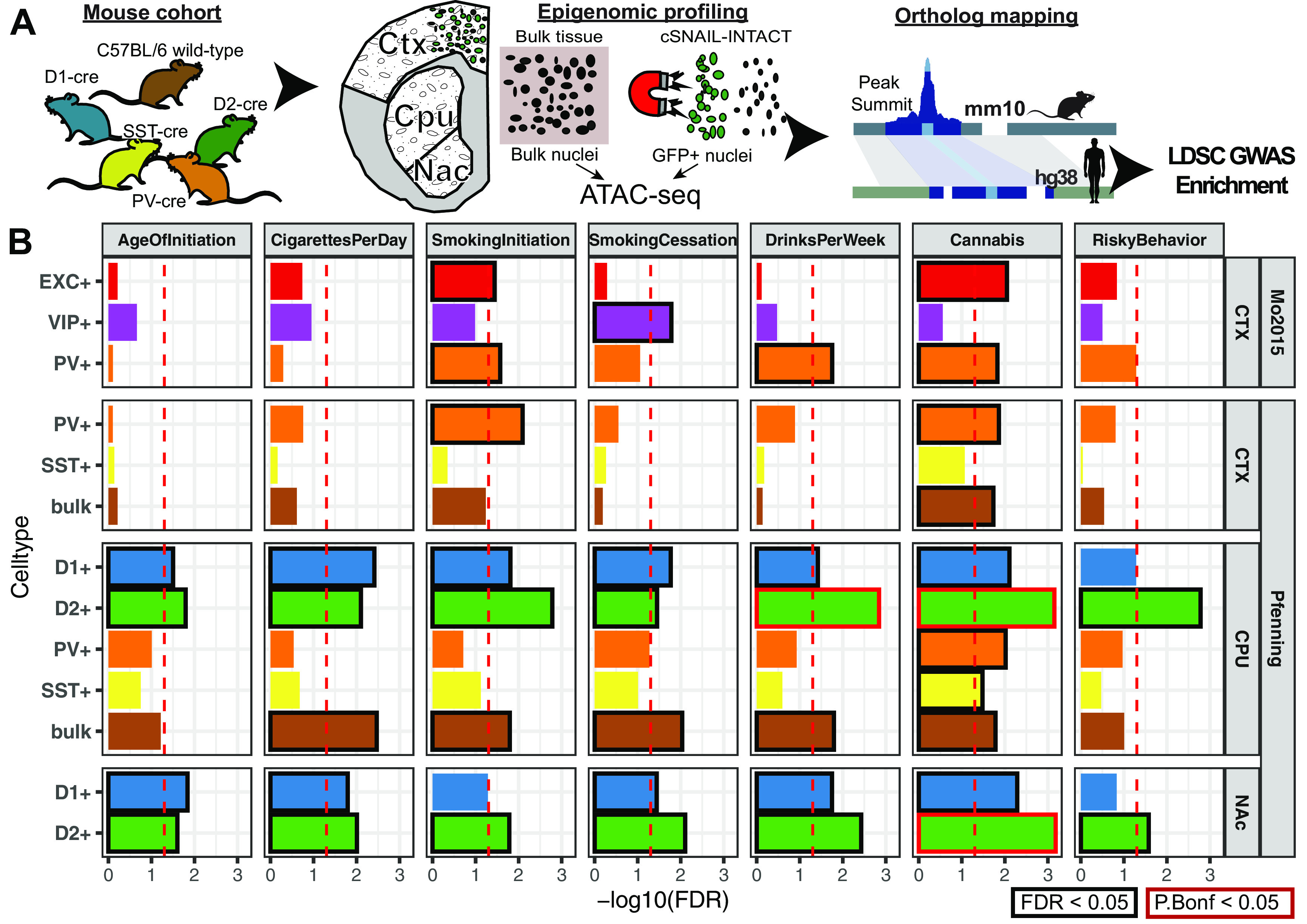
Cell type-specific enrichment of substance use traits are conserved in mouse-human orthologous OCRs. ***A***, Experimental design to map human orthologous regions from mouse ATAC-seq of bulk cortex (CTX), dorsal striatum (CPU), and NAc of cSNAIL nuclei of D1-cre, A2a-cre, PValb-2a-cre, and SST-cre mice. cSNAIL ATAC-seq experiments report enriched (+) nuclei populations. ***B***, Stratified LD score regression finds enrichment of substance use and risky behavior traits for brain region- and cell type-specific ATAC-seq open chromatin profiles of mouse brain. Replication of enrichment is shown using INTACT-enriched OCRs from Mo et al. (2015) of cortical excitatory (EXC^+^), VIP interneuron (VIP^+^), and PV interneuron (PV^+^). Enrichments that are enriched at FDR ≤ 0.05 are plotted with black outlines and Bonferroni *p* value ≤ 0.05 with red outlines. FDR-adjusted *p* value was performed across all mouse-human ortholog GWAS enrichment across Figure 5.

In order to further interrogate the different neuronal subtypes that comprise the enrichment of addiction-associated genetic variants in OCR sets measured by Fullard et al. (2018), Corces et al. (2020), and Lake et al. (2018) (Figs. 2 and 3), we performed targeted epigenomic experiments in mouse on isolated neuronal subtypes from key brain regions of the reward circuit: frontal cortex (CTX), caudoputamen (CPU), and the NAc. We isolated nuclei from specific cell types for ATAC-seq using a modified version of the INTACT approach (Mo et al., 2015) called cSNAIL (see Experimental design). cSNAIL-INTACT was applied to isolate nuclei marked by *Pvalb*, *Sst*, *Drd1*, and *Adora2a* in *cre-driver* lines using a shortened form of the *Sun1-Gfp* fusion protein packaged with AAV-PHP.eb and delivered through retro-orbital injection (see Fig. 5*A*). We show that cell type-targeting provided markedly distinct genome-wide ATAC-seq profiles compared with bulk tissue ATAC-seq alone (Fig. 4*A*). cSNAIL ATAC-seq specifically captured nuclei with increased accessibility around the marker gene that was driving *Cre* recombinase expression (Fig. 4*B*). Accessibility around cSNAIL ATAC-seq TSSs strongly correlated with matched pseudobulk gene expression in the same cell type and tissue (see Materials and Methods, both Pearson and Spearman correlation P_bonf_ < 2 × 10^−16^_;_ Fig. 4*C*,*D*). We applied HALPER, an approach that leverages reference-free multispecies genome alignments to produce 1-1 contiguous CRE orthologs (Zhang et al., 2020), to reliably map ∼70% of mouse neuronal subtype OCRs to their human orthologs in the hg38 human reference genome (see Statistical methods) for LDSC regression GWAS analysis.

Our GWAS enrichment analysis of human orthologs from mouse OCRs (mouse-human orthologs) measured in various neuronal subtypes and bulk tissue (Fig. 5*B*) show that genetic variants associated with SmokingInitiation and Cannabis shared enrichment in cortical EXC and PV neuron OCRs from both Mo et al. (2015) (SmokingInitiation- CTX PV+ FDR = 2.6E-02, Cannabis- CTX PV+ FDR = 8.9E-03, SmokingInitiation- CTX EXC+ FDR = 3.6E-02, Cannabis- CTX EXC+ FDR = 1.4E-02) and the current study (Pfenning data; SmokingInitiation- CTX PV+ FDR = 2.6E-02, Cannabis- CTX PV+ FDR = 1.3E-02). Genetic variants associated with Cannabis further enriched in CTX bulk tissue OCRs (FDR = 1.8E-02), which could be attributed to signal from cortical EXC and PV neuron populations. Cortical PV neuron OCRs further enriched with genetic variants associated with DrinksPerWeek (FDR = 1.7E-02). SmokingCessation-associated genetic variants distinctly enriched in cortical VIP neuron OCRs (FDR = 1.6E-02).

Within neuronal subtypes from CPU and NAc, we found enrichment of genetic variants associated with all measured addiction-associated traits in CPU D2 MSN (AgeOfInitiation FDR = 1.6E-02, CigarettesPerDay FDR = 7.9E-03, SmokingInitiation FDR = 1.6E-03, SmokingCessation FDR = 3.5E-02, DrinksPerWeek FDR = 1.4E-03, Cannabis FDR = 7.1E-04, Cannabis P_Bonferroni_ = 1.9E-02, RiskyBehavior FDR = 2.6E-02) and NAc D2 MSN OCRs (AgeOfInitiation FDR = 2.5E-02, CigarettesPerDay FDR = 9.8E-03, SmokingInitiation FDR = 1.6E-02, SmokingCessation FDR = 7.9E-03, DrinksPerWeek FDR = 3.7E-03, Cannabis FDR = 6.6E-04, Cannabis P_Bonferroni_ = 1.6E-02, RiskyBehavior FDR = 1.6E-03). Genetic variants associated with all measured traits, excluding SmokingInitiation and RiskyBehavior, all enriched in NAc D1 MSN OCRs (AgeOfInitiation FDR = 1.4E-02, CigarettesPerDay FDR = 1.6E-02, SmokingCessation FDR = 3.6E-02, DrinksPerWeek FDR = 1.7E-02, Cannabis FDR = 5.1E-03). CPU D1 MSN OCRs were enriched with genetic variants associated with all measured traits, excluding RiskyBehavior (AgeOfInitiation FDR = 3.1E-02, CigarettesPerDay FDR = 3.8E-03, SmokingInitiation FDR = 1.5E-02, SmokingCessation FDR = 1.6E-02, DrinksPerWeek FDR = 3.7E-02, Cannabis FDR = 7.8E-03). We found that CPU bulk tissue OCRs were enriched with genetic variants associated with all measured addiction-associated traits, excluding AgeOfInitiation and RiskyBehavior (CigarettesPerDay FDR = 3.4E-03, SmokingInitiation FDR = 1.6E-02, SmokingCessation FDR = 9.2E-03, DrinksPerWeek FDR = 1.6E-02, Cannabis FDR = 1.6E-02). Distinctly, CPU PV+ and SST+ neuron OCRs enriched with genetic variants associated with Cannabis (CPU PV+ FDR = 9.5E-03, CPU SST+ FDR = 3.3E-02).

**Figure 6.**
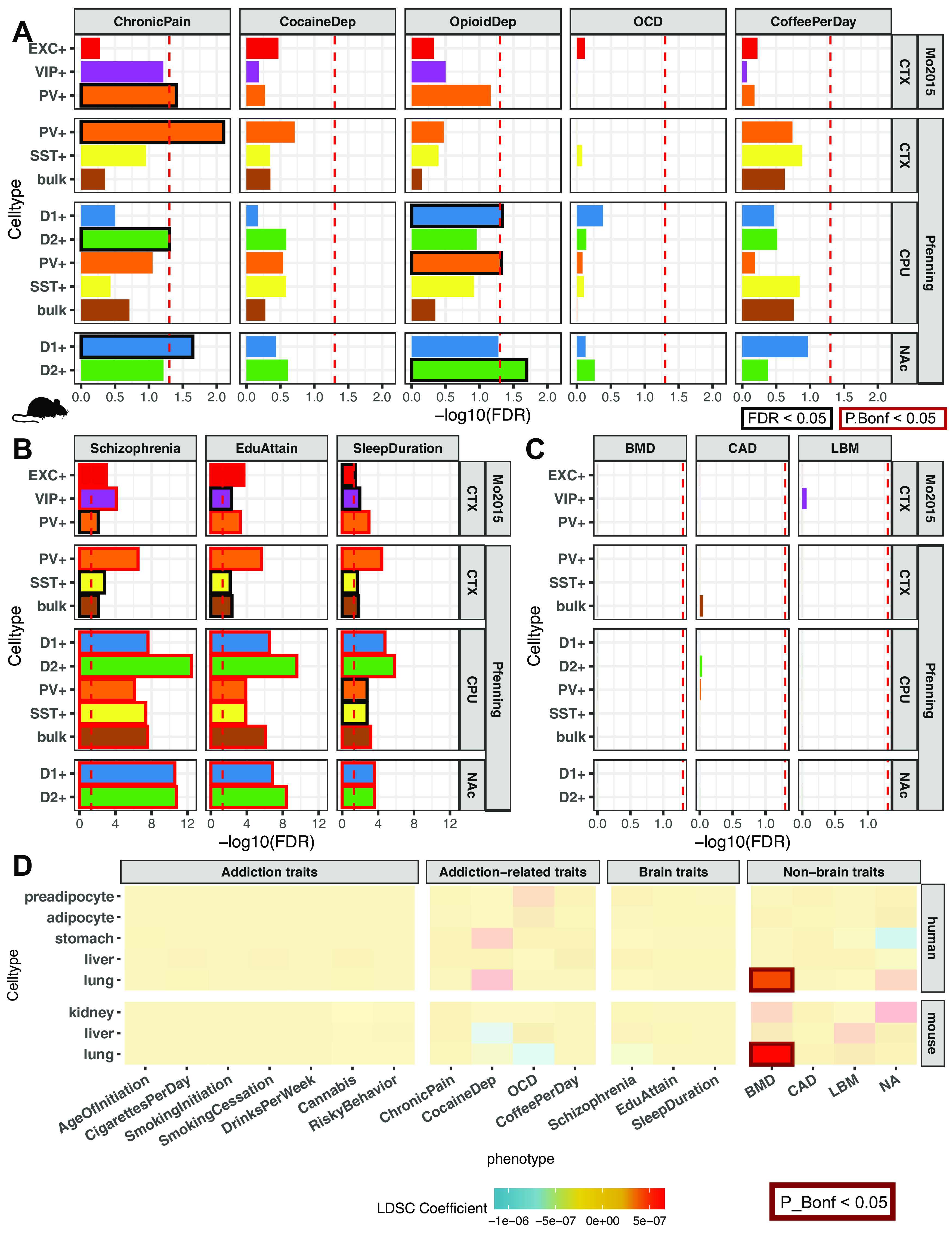
GWAS enrichment in addiction- and non–addiction-related traits using mapped mouse orthologs of tissue- and cell type-specific OCRs. GWAS enrichment plots with FDRs in human orthologous regions mapped from mouse ATAC-seq of bulk cortex (CTX), dorsal striatum (CPU), and NAc or cSNAIL nuclei of D1-cre, D2-cre, and PV-cre mice. cSNAIL ATAC-seq experiments report both enriched (+) and de-enriched (–) nuclei populations. Enrichments that are enriched at FDR < 0.05 are plotted with black outlines. Replication of enrichment is shown using INTACT-enriched OCRs from Mo et al. (2015) of cortical excitatory (EXC^+^), VIP interneuron (VIP^+^), and PV interneuron (PV^+^). ***A***, GWAS enrichment of addiction- or substance use-associated traits: multisite chronic pain (ChronicPain), cocaine dependence (CocaineDep), opioid dependence (OpioidDep), diagnosis of OCD, and cups of coffee drank per day (CoffeePerDay). The GWASs for OCD, opioid dependence, and cocaine dependence are reportedly underpowered to detect genetic liability for these traits (*N*_case_< 5000). ***B***, GWAS enrichment in well-powered brain-related traits shows cell type- and region-specific enrichment: educational attainment (EduAttain), schizophrenia risk (Schizophrenia), and habitual sleep duration (SleepDuration). ***C***, GWAS enrichment in non–brain-associated traits does not show cell type- or region-specific enrichment: heel BMD, CAD, and LBM. ***D***, Heatmap of LDSC regression coefficients of GWAS enrichment for all measured GWASs in nonbrain OCRs from human or mouse-human mapped orthologs. Tissues for which OCRs are significantly enriched (FDR < 0.05, black; Bonferroni *p* value ≤ 0.05, red) with GWAS variants are outlined with a bolded box.

Corresponding to our analysis of human brain OCRs, we also confirmed the specificity of mouse-human orthologous CRE enrichments for genetic variants associated with addiction-related, brain-related, and non–brain-related traits (Fig. 6). We found enrichments of genetic variants associated with ChronicPain in cortical PV neuron OCRs from both Mo et al. (2015) (FDR = 3.9E-02) and the current study (Fig. 6*A*; FDR = 7.9E-03). Within striatal cell types, we found that CPU D2 and NAc D1 MSN OCRs were enriched for genetic variants associated with ChronicPain (CPU D2+ FDR = 4.9E-02, NAc D1+ FDR = 2.2E-02). In contrast, CPU D1 and NAc D2 MSN OCRs were enriched for genetic variants associated with OpioidDep (CPU D1+ FDR = 4.5E-02, NAc D2+ FDR = 2.0E-02). Genetic variants associated with OpioidDep also enriched in CPU PV OCRs (FDR = 4.7E-02). Schizophrenia-, EduAttain-, and SleepDuration-associated genetic variants all enriched in OCRs of all measured cell types (Fig. 6*B*). None of these mouse-human orthologs enriched for genetic variants associated with non–brain-related traits: BMD, CAD, and LBM (Fig. 6*C*). We validated that our approach to map OCRs from mouse to human did not bias enrichment to brain traits by performing GWAS enrichment on OCRs from mouse nonbrain tissues (kidney, liver, and lung) (Fig. 6*D*). As expected, we did not find an enrichment for genetic variants associated with a brain-related trait. We did find that mouse-human orthologs of lung OCRs enrich for BMD (FDR = 8.3E-03), which concords with the enrichment of human lung OCRs.

### CNN models of mouse cell type-specific CRE activity refine human NeuN^+^ OCRs for GWAS enrichment

The genetic tools available for mouse research allowed us to isolate the nuclei of specific neuronal subtypes and generate deep open chromatin profiles at greater cellular resolution. However, a lack of directly measured mouse-human conservation could lead to false negatives and false positives in the cell type specificity of CREs at specific loci that add noise to GWAS comparisons. To leverage the strengths of the mouse and human approaches, we developed a procedure to predict the neuronal subtype specificity of human OCRs using machine learning models trained in mouse. The OCR profile of each neuronal subtype is largely a result of a developmental cascade of transcription factors that cooperatively recognize and bind to specific sequence elements in the genome, resulting in a neuronal subtype-specific open chromatin profile (Spitz and Furlong, 2012). These complex combinations of sequence features comprise a regulatory code that links genome sequence to neuronal subtype-specific open chromatin. This regulatory code can be effectively learned using CNNs and has been demonstrated to be highly conserved between mouse and human (Zhou and Troyanskaya, 2015; L. Chen et al., 2018).

**Figure 7.**
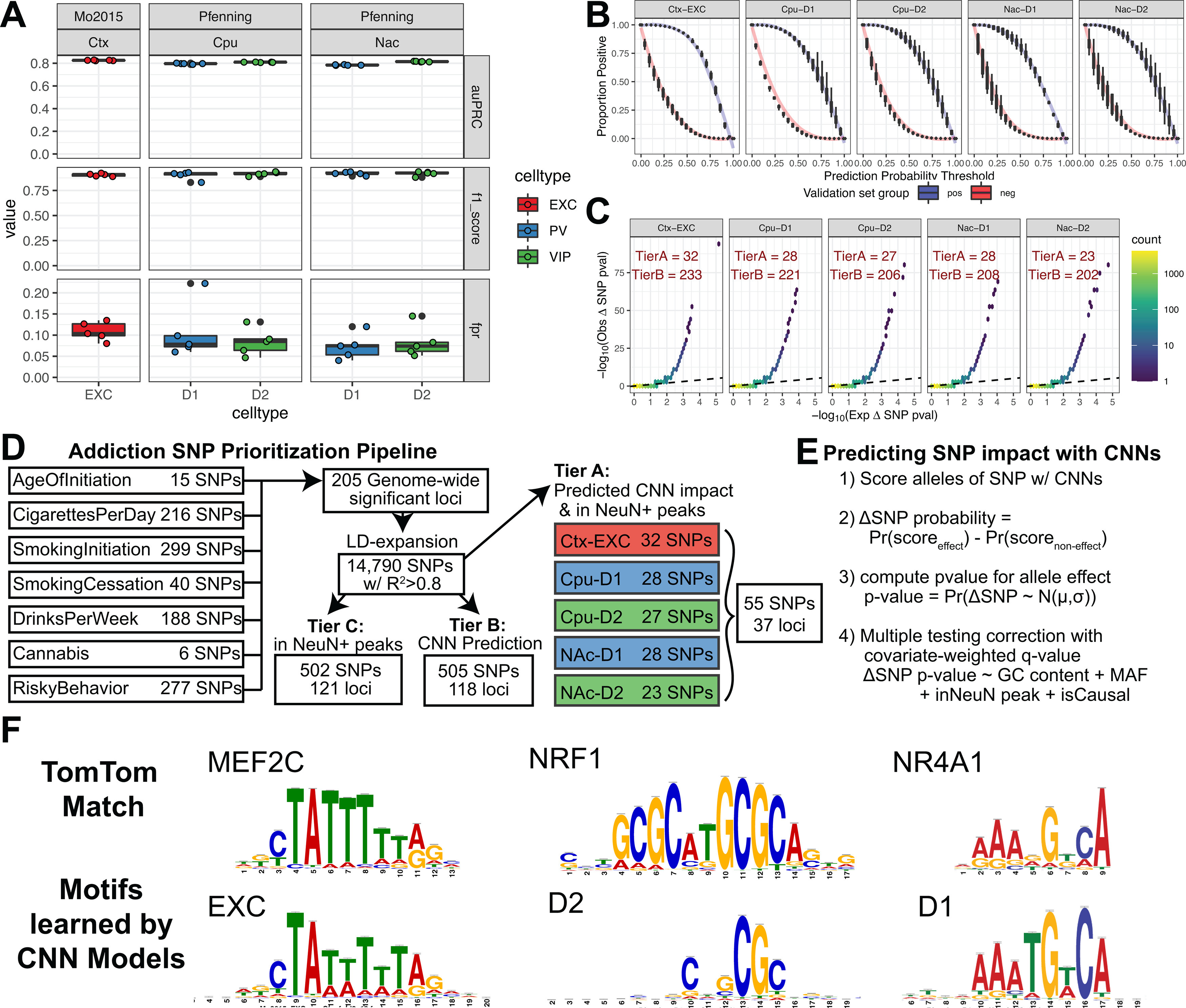
CNN model performance and selection of candidate functional SNPs. ***A***, Performance metrics for CNN models show high specificity on the test sets of positive peaks or 10× nucleotide content-matched negatives. Test set performance metrics are reported for auPRC, F1 score (using threshold = 0.5), and false positive rates across all possible thresholds (see Materials and Methods). Models were trained on IDR peaks of mouse cortical EXCs (Ctx-EXC) and D1 and D2 MSNs from CPU and NAc. ***B***, The models best discriminate the proportion of positives and negative sequences at a threshold of 0.5. Plots represent the proportion of positives (blue) or negatives (red) that are called “positive” across CNN output thresholds from 0 to 1 averaged across folds for each set of CNN models. ***C***, Quantile-quantile plots of *p* values of calibrated ΔSNP probabilities (see Materials and Methods) from a normal distribution after centering by the mean and scaling by the SD of δ SNP probabilities across all SNPs (*n* = 14,790 SNPs) for each set of CNN models. A hexbin plot was used to visualize overplotting, where every hexagon is colored by the number of SNPs in that bin. Black dotted line indicates the equality line *y* = *x*. The number of significant SNPs at FDR *q* value < 0.05 at Tier A or B are reported for each cell type and tissue (see Materials and Methods). ***D***, Schematic to select for predicted causal impact addiction-associated GWAS SNPs. The pipeline begins with SNPs across addiction-associated GWASs aggregated to 205 nonoverlapping GWAS loci across 14,790 SNPs after LD expansion to include those in LD *R*^2^ > 0.8 (Extended Data Fig. 7-2). SNPs are further prioritized into three tiers. Tier C includes SNPs that only overlap Fullard et al. (2018), NeuN^+^ ATAC-seq peaks. Tier B includes SNPs with only predicted significant differential allelic impact on CNN-predicted CRE activity at *q* value < 0.05. Tier A includes SNPs satisfying both criteria (see Materials and Methods). ***E***, Outline of predicting differential CRE activity between alleles using calibrated CNN probabilities of CRE activity while controlling for FDR with informative covariates (see Materials and Methods). ***F***, Example motif matches from Extended Data Figure 7-1 of TomTom known transcription factor consensus motifs and the learned important features in CNN models for cortical excitatory and striatal D1 and D2 MSNs.

The concordant pattern of enrichment for addiction-associated genetic variants in human and mouse-human orthologous OCRs suggested that risk variants may affect the regulatory activity of neuronal subtypes that are conserved between human and mouse. We therefore devised and trained a collection of CNN binary classification models to learn the genome sequence features that distinguish OCRs for cortical EXC neurons, striatal D1 MSNs, and striatal D2 MSNs (Zhou and Troyanskaya, 2015; Kelley et al., 2016, 2018; L. Chen et al., 2018). For each set of reproducible OCRs from the mouse INTACT and cSNAIL data, we trained models to predict the reproducible peaks from ∼10 times the number of nucleotide content-matched negative sequences (see Materials and Methods). Our models made confident predictions on held-out test sequences as reported by high F1 scores, high auPRCs (Fig. 7*A*), and low false positive rates at a threshold of 0.5 (Fig. 7*B*). These models reproducibly learned transcription factor motif families that are enriched in human neuronal subtypes of cortex (*MEF2*, *JUN*) and striatum (*POU*, *NRF1*, *ZFHX3*), as previously reported by Fullard et al. (2018) (Fig. 7*F*; Extended Data Fig. 7-1).

10.1523/JNEUROSCI.2534-20.2021.f7-1 Extended Data Figure 7-1 TomTom matches with motifs learned by CNN models in each cell type and fold to contribute to a strong positive prediction. Learned important features were interpreted by DeepSHAP and clustered into unique seqlets by TF-Modisco (see Materials and Methods). Download Figure 7-1, XLSX file.

10.1523/JNEUROSCI.2534-20.2021.f7-2 Extended Data Figure 7-2 Addiction-associated genetic variants annotated with cell type and brain region functional markers. Addiction-associated genetic variants from the main seven GWAS (Figure 7) that were scored by CNN models along with computed raw CNN scores, predicted probability active, and ΔSNP probabilities, and tier of predicted candidate causal SNP. Each entry is recorded for a distinct SNP, predicted CNN model, and GWAS trait. Additional columns reporting are annotated by FUMA (Watanabe et al., 2017) and CAUSALdb (J. Wang et al., 2020). SNPs are annotated in this study to overlap with human NeuN^+^ OCRs (Fullard et al., 2018). A complete legend describing column headers is in the first sheet of the table. Download Figure 7-2, XLSX file.

**Figure 8.**
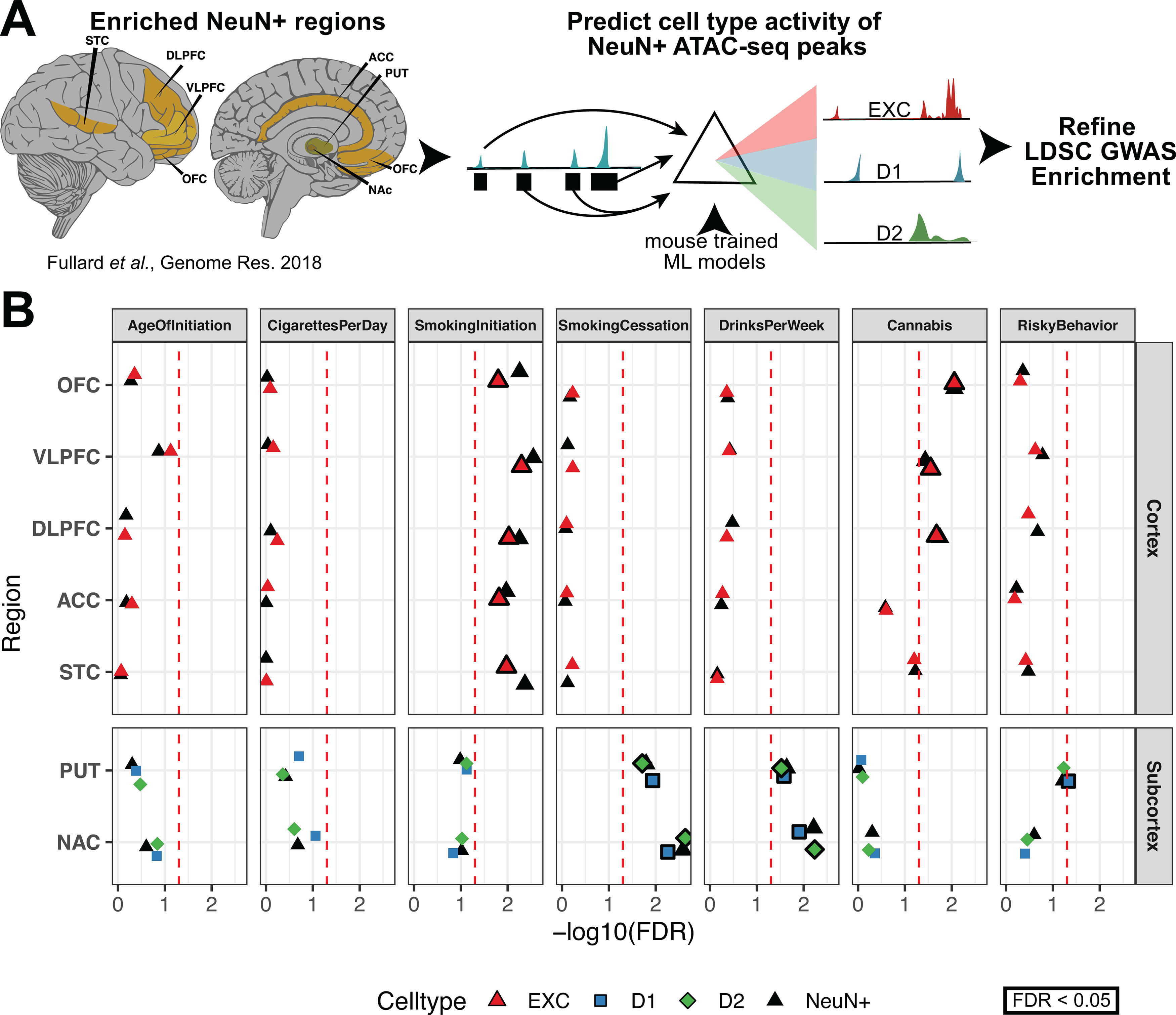
Cell type-specific CNN models refine human NeuN^+^ enrichments for substance use genetic risk GWASs. ***A***, Schematic to predict cell type-specific activity of NeuN^+^ ATAC-seq peaks enriched from brain regions assayed in Fullard et al. (2018) using CNN models trained on mouse cell type-specific ATAC-seq peaks. CNN-predicted OCRs are used as input for computing GWAS enrichment. ***B***, Stratified LD score regression of addiction-associated traits in Fullard et al. (2018). NeuN^+^ OCRs are predicted to be cell type-specific by machine learning models of open chromatin. Cell types are colored by the source mouse cell type-specific OCRs from ***A***. Original enrichments from Figure 5*A* are reproduced in black. Larger, bolded points are significant for FDR < 0.05 (red dotted line).

We reasoned that NeuN^+^ OCR signal, which is comprised of OCR signals from several neuronal subtypes, can be parsed into its component cell types by CNNs that are trained to predict OCR activity in those component cell types. This enables the study of human addiction genetics at a cell type-level resolution from high-quality tissue-level open chromatin profiles. To discern whether NeuN^+^ OCR enrichments for addiction-associated genetic variants come from the same cell types observed in Figure 2, we applied our trained CNN models to predict whether bulk cortical or striatal NeuN^+^ OCRs have activity in either cortical EXC or striatal D1 and D2 neurons, respectively (Fig. 8*A*). We did not conduct these analyses for PV, SST, or VIP interneurons because they comprise a much lower percentage of cortical and striatal neurons than the other neuron types (Beaulieu, 1993; Lefort et al., 2009). We ran LDSC regression (Finucane et al., 2018) GWAS enrichments on the sets of NeuN^+^ OCRs predicted to be specific to cortical EXC, striatal D1, and striatal D2 neurons. Genetic variants associated with SmokingInitiation, which initially were enriched for occurring in OCRs of various NeuN^+^ frontal cortical areas (Fig. 2*A*), were enriched for occurring in NeuN^+^ OCRs predicted to be active in EXC neurons (Fig. 8*B*; VLPFC FDR = 5.0E-03, DLPFC FDR = 9.4E-03, STC FDR = 1.0E-02, ACC FDR = 1.5E-02, OFC FDR = 1.5E-02). Genetic variants associated with Cannabis, which were enriched for occurring in NeuN^+^ cortical OCRs (Fig. 2*A*), were also enriched for occurring in NeuN^+^ OCRs predicted to be active in EXC neurons (OFC FDR = 8.7E-03, DLPFC FDR = 1.7E-02, VLPFC FDR = 3.6E-02). The enrichments of excitatory cortical cell type-specific OCRs for SmokingInitiation and Cannabis associated genetic variants agree with the results from the analysis of the Fullard et al. (2018), Corces et al. (2020), and Lake et al. (2018) OCR datasets (Fig. 2). Genetic variants associated with SmokingCessation and DrinksPerWeek, which were enriched for occurring in PUT and NAc NeuN^+^ OCRs (Fig. 2*A*), were enriched for occurring in OCRs predicted to be active in D1 and D2 MSNs of PUT and NAc (SmokingCessation-D2 NAc FDR = 2.3E-03, SmokingCessation-D1 NAc FDR = 5.4E-03, SmokingCessation-D1 PUT FDR = 1.1E-02, SmokingCessation-D2 PUT FDR = 1.9E-02, DrinksPerWeek-D2 PUT FDR = 5.8E-03, DrinksPerWeek-D1 PUT FDR = 1.2E-02, DrinksPerWeek-D1 NAc FDR = 2.6E-02, DrinksPerWeek -D2 NAc FDR = 2.9E-02). Thus, our new framework that we applied to these addiction-related traits (outlined in Fig. 8*A*) refines addiction genetic risk signal to neuronal subtypes within specific brain regions. This framework can be applied to CREs from any tissue-cell type combination for which bulk tissue open chromatin measurements are available from human and cell type open chromatin measurements are available from another vertebrate (L. Chen et al., 2018; Minnoye et al., 2020).

### CNN models predict allele-specific activity of addiction-associated GWAS SNPs in neuronal subtypes

**Figure 9.**
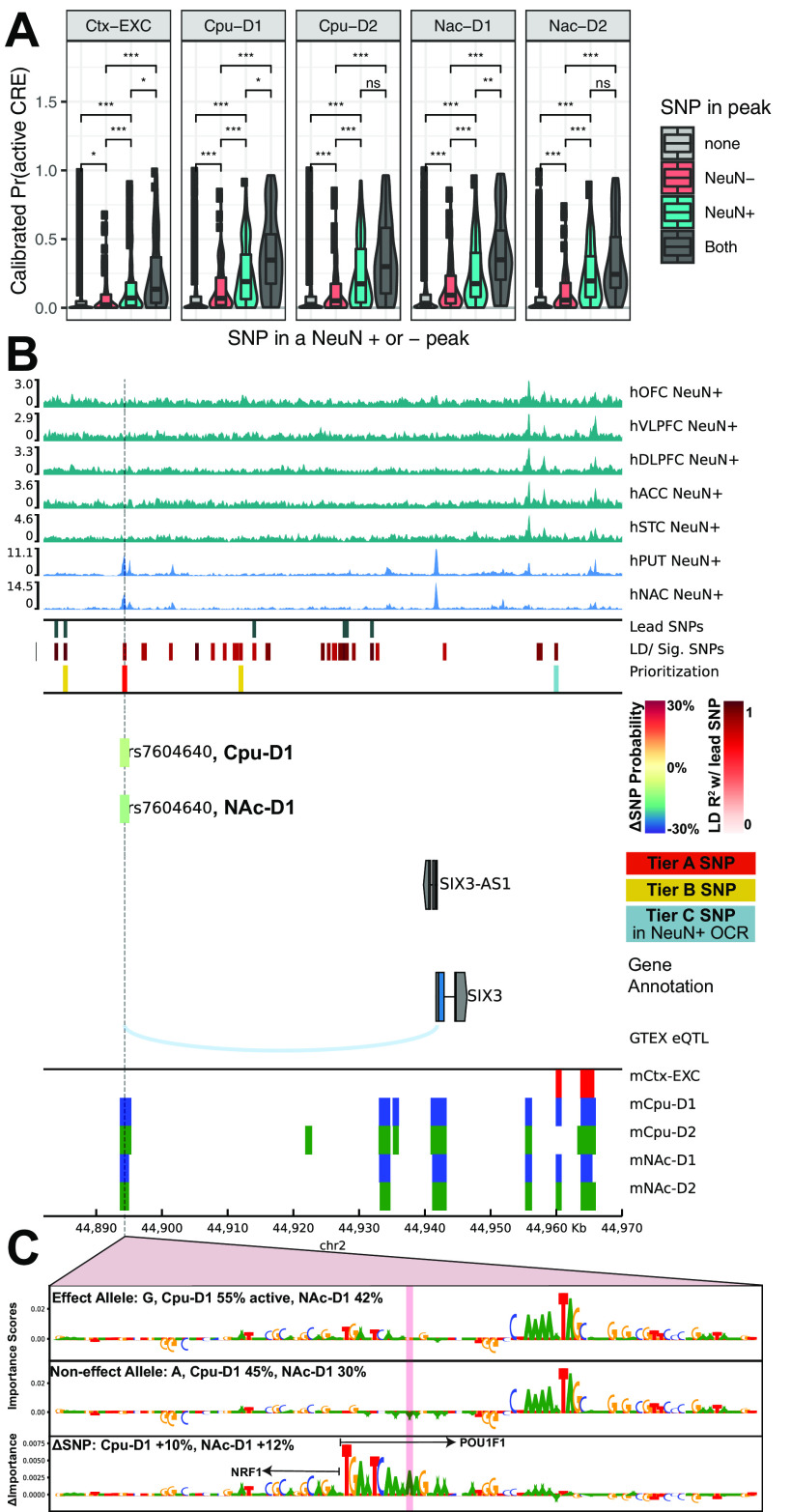
CNN models for predicting cell type-specific open chromatin predict activity of addiction GWAS SNPs. ***A***, Cell type activity predicted probability active by each set of CNN models of cell type activity for genome-wide significant SNPs and off-lead SNPs in LD *R*^2^ > 0.8 with the lead SNPs. Activity scores for SNPs are stratified by overlap with Fullard et al. (2018) cortical or striatal NeuN^+^ (teal), NeuN^–^ peaks (salmon), both (dark gray), or neither (light gray). Significance symbols indicate Bonferroni-adjusted *p* values from two-tailed *t* tests for *N* = 18 possible pairwise comparisons: *p < 0.05/*N*; **p < 0.01/*N*; ***p < 0.001/*N*. ***B***, Locus plot for candidate SNPs with predicted function SNP impact in cortical excitatory and striatal D1, and D2 MSN cell types. Genome tracks from top to bottom: human (h) NeuN^+^ MACS2 ATAC-seq fold change signal of cortical and striatal brain regions enriched in Figure 5*A*. SNP tracks show lead SNPs from seven addiction-associated GWASs and the SNPs either in LD with the lead SNPs (Lead SNPs) or independently significant SNPs (LD/Sig. SNPs). Each SNP is colored by increasing red intensity that indicates the degree of LD with a lead SNP. Prioritized candidate causal SNPs by predicted differential cell type activity and overlap with Fullard et al. (2018). NeuN^+^ OCRs are plotted as follows: red represents Tier A; yellow represents Tier B; teal represents Tier C (see Materials and Methods). Tier A SNP rs7604640 is predicted to have a strong ΔSNP effect by CPU-D1 and NAc-D1 CNN models, and the bars are colored by the % change in probability active. Gene annotation tracks plot GENCODE genes from the GRCh38 build. eQTL link tracks of FDR-significant GTEX *cis*-eQTL from cortical or striatal brain regions, and orthologs of mouse (m) putative CREs mapped from excitatory or striatal neuronal subtypes measured by cSNAIL ATAC-seq. Cell type colors label cortical EXCs (EXC; red), D1 MSNs (D1; blue), or D2 MSNs (D2; green). ***C***, Representative importance scores of 50 bp flanking either side of the SNP rs7604640 that measure relative contribution of that sequence being active in D1 MSNs. CNN importance score interpretations are shown for effect and non-effect alleles, and the difference in importance scores reveals the relatively more important DNA motif in the effect allele that matches consensus POU1F1 motif overlapping the rs7604640 SNP. The model interprets this POU1F1 motif and a nearby NRF1 motif as contributing to the effect allele having more activity in D1 MSNs.

Last, we applied our CNN models to screen addiction-associated genetic variants for predicted functional activity in EXC, D1, and D2 neuronal subtypes. CNN-based approaches have been demonstrated to fine-map dense risk loci and select candidate causal genetic variants (Alipanahi et al., 2015; Zhou and Troyanskaya, 2015; Kelley et al., 2016, 2018; Corces et al., 2020), yet none has been applied in the context of addiction-associated genetic risk or in the cell types that we have assayed. We identified 14,790 unique SNPs that were collected across the seven addiction-associated GWASs to score for differential neuronal subtype OCR activity (see Materials and Methods). We expect that many SNPs reported from GWASs are significantly associated with traits because of LD rather than being the true causal variant. When scored with our CNN models, the 96.2% of addiction-associated SNPs that either do not lie in any OCR or in only NeuN^–^ OCRs have low probabilities of being active in excitatory, D1, or D2 neuronal subtypes. We also found that these SNPs have significantly lower predicted probabilities of activity than the remaining 3.8% of addiction-associated SNPs in any NeuN^+^ OCR (P_Bonferroni_ < 0.05; Fig. 9*A*). We then predicted the probability of activity for both the effect and non-effect alleles and estimated the differential impact of the alleles to fine-map candidate causal effect SNP and target neuronal subtypes and tissues. Most SNPs do not have predicted differential allelic activity (δ SNP) in a neuronal subtype, while a handful of SNPs have larger differential activity that deviate from a normal distribution when visualized on quantile-quantile plots (Fig. 7*C*; see Materials and Methods). We outline in Figure 7*D* an approach to prioritize the candidate causal SNPs by two lines of evidence: (1) a predicted differential neuronal subtype OCR activity with large effect size that is controlled for FDR (*q* < 0.05; see Materials and Methods); and (2) having physical overlap with measured human NeuN^+^ OCR in Fullard et al. (2018) (Fig. 7*D*). We are able to prioritize 55 SNPs spanning 37 loci to Tier A that both have significant predicted ΔSNP probability effect and overlap Fullard et al. (2018), NeuN^+^ OCR, 505 SNPs to Tier B that only have predicted ΔSNP probability effect, and 502 SNPs to Tier C that overlap NeuN^+^ open chromatin but do not have a predicted significant ΔSNP probability effect (Extended Data Fig. 7-2).

One such SNP from Tier A, rs7604640, lies in human NeuN^+^ open chromatin specific to striatum 46 kb upstream of the *SIX3* locus on chromosome 2. rs7604640 overlaps human orthologs of mouse OCRs in only D1 and D2 neurons, and we predict the effect allele of rs7604640 has an increased probability of open chromatin activity in D1 OCRs of the striatum compared with the non-effect allele (Fig. 9*B*). rs7604640 is one of many off-lead SNPs identified in the SmokingInitiation GWAS (P_GWAS_ = 3.04 × 10^−12^) and is in high LD with the SNP rs163522 (*R*^2^ = 0.856, P_GWAS_ = 1.11 × 10^−11^), which is independently significant from the lead SNP, rs1004787 (*R*^2^ = 0.630, P_GWAS_ = 5.27 × 10^−17^). rs7604640 was reported by HaploRegv4 to overlap a POU1F1 motif (Ward and Kellis, 2016), which our D1 models predict to contribute toward increased probability of being active in D1 MSNs (Fig. 9*C*). Furthermore, this SNP is a known *cis*-eQTL for the antisense *SIX3-AS1* gene in striatal regions from the Genotype-Tissue Expression (GTEX) project (GTEx Consortium, 2013, 2015, 2017; Melé et al., 2015). Antisense gene expression is one mechanism of regulating their sense gene (Pelechano and Steinmetz, 2013; Barman et al., 2019), and deletion of the gene *SIX3* has been shown to inhibit development of D2 MSNs (Xu et al., 2018). Together, this evidence formulates the hypothesis that common genetic variant rs7604640 has D1 MSN-specific, allelic impact on open chromatin activity in a mouse-human conserved putative CRE regulating the MSN regulator *SIX3*.

**Figure 10.**
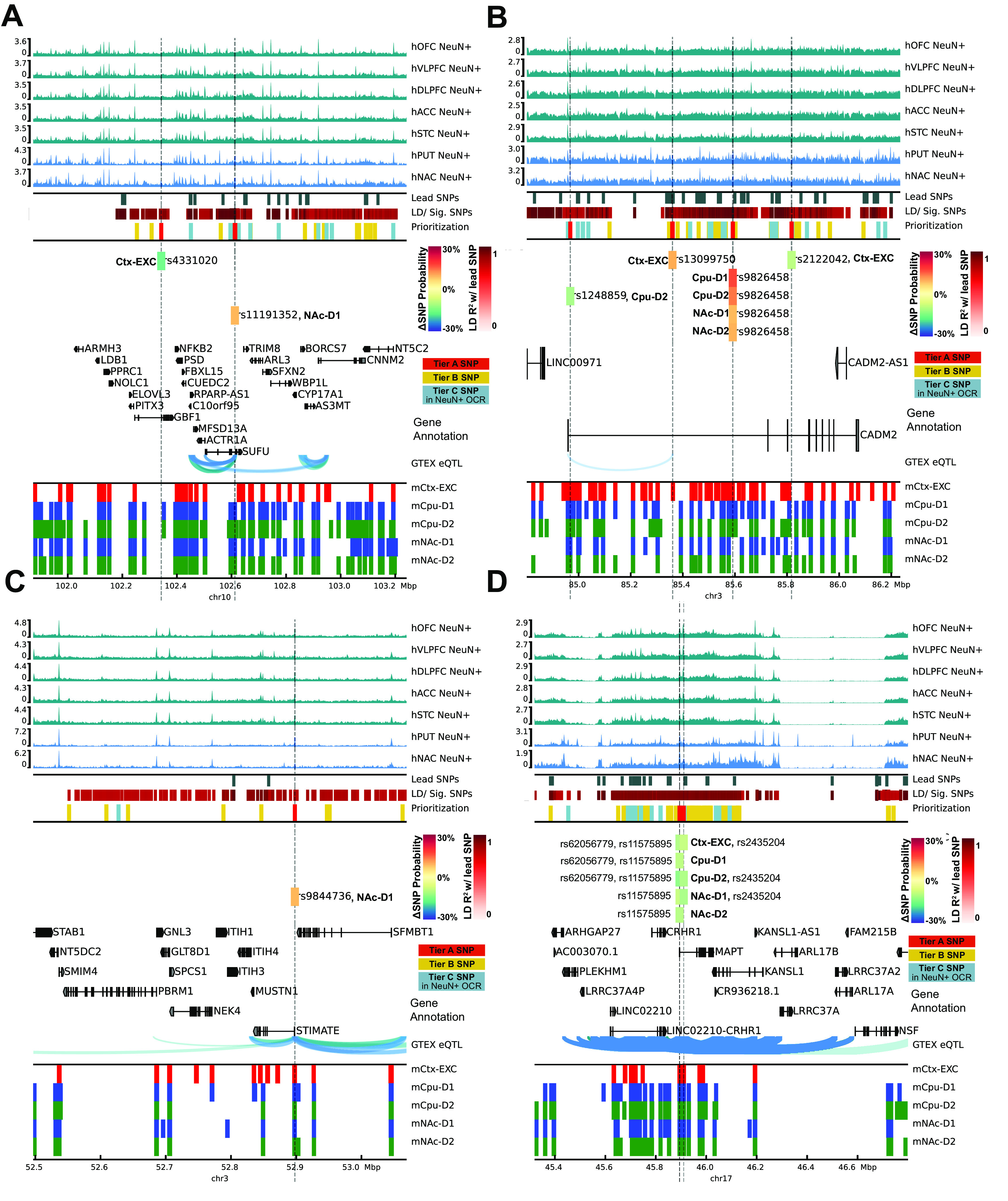
Locus plots of addiction-associated SNPs predicted to act in striatal and cortical cell types. Locus plots are located on human (***A***) chr10, (***B*** and ***C***) chr3, and (***D***) chr17. Locus plot across four additional loci with Tier A SNPs with predicted function SNP impact in cortical excitatory and striatal D1 and D2 MSN cell types. Genome tracks from top to bottom: human (h) NeuN^+^ MACS2 ATAC-seq fold change signal of cortical and striatal brain regions enriched in Figure 5*A*. SNP tracks plot lead SNPs aggregated across seven addiction-associated GWASs, the SNPs in LD with the lead SNPs (Lead SNPs), or independently significant SNPs (LD/Sig. SNPs). Each SNP is colored red, increasing in intensity by the degree of LD with a lead SNP. Prioritized candidate causal SNPs by predicted differential cell type activity and overlap with Fullard et al. (2018). NeuN^+^ OCRs are plotted as follows: red represents Tier A; yellow represents Tier B; teal represents Tier C (see Materials and Methods). Tier A SNP rs7604640 is predicted to have strong ΔSNP effect by CPU-D1 and NAc-D1 CNN models, and the bars are colored by the % change in probability active. Gene annotation tracks plot GENCODE genes from the GRCh38 build. eQTL link tracks of FDR-significant GTEX *cis*-eQTL from cortical and striatal brain regions, and orthologs of mouse (m) putative CREs mapped from excitatory or striatal neuronal subtypes measured by cSNAIL ATAC-seq. NeuN^+^ ATAC-seq tracks and eQTL links are colored by source brain region as cortical (teal) or striatal (blue). Cell type colors label cortical EXCs (EXC; red), D1 MSNs (D1; blue), or D2 MSNs (D2; green).

In addition to rs7604640, we report four loci with 1-4 candidate SNPs each in Tier A that may be putative causal SNPs with cell type-specific activity in addiction-associated traits (Fig. 10). The SNPs in these loci all have reported eQTL in frontal cortex or striatum tissues from GTEx, and they overlap corresponding NeuN^+^ OCRs and mouse-human orthologous OCRs. In some cases, our prioritized Tier A SNPs were predicted to have ΔSNP effects (see Materials and Methods) in only striatal MSNs, showcasing our framework's ability to predict cell type-specific impact. These SNPs include rs11191352 (P_SmokingInitiation_ = 2.12 × 10^−7^; Fig. 10*A*), rs9826458 (P_RiskyBehavior_ = 4.36 × 10^−21^, P_SmokingInitiation_ = 1.21 × 10^−14^; Fig. 10*B*), and rs9844736 (P_RiskyBehavior_ = 3.04 × 10^−7^, P_SmokingInitiation_ = 3.58 × 10^−7^; Fig. 10*C*). In a few cases, our models predicted SNPs to have strong ΔSNP effects across both cortical excitatory and striatal cell types. These include two SNPs in the highly pleiotropic *MAPT-CRHR1* locus that are 152 bp apart and in perfect LD with each other, rs11575895 and rs62056779 (Fig. 10*D*). The prioritized SNPs in the *MAPT-CRHR1* locus are genome-wide significant for 5 of the 7 addiction-associated traits (Extended Data Fig. 7-1), and the locus has been implicated in other neuropsychiatric traits, such as Alzheimer's disease (Hoffman et al., 2019; Corces et al., 2020; Ramamurthy et al., 2020). We provide the summary of CNN predictions in these reported loci across all 14,790 analyzed SNPs along with the accompanying annotations that we incorporated into our prioritization of candidate causal SNPs and their predicted cell types (Extended Data Fig. 7-1).

## Discussion

In this study, we demonstrate the first analyses integrating neuronal subtype OCRs across human and mouse brain epigenomics using CNN models to select candidate addiction-associated SNPs acting at putative neuronal subtype-specific CREs. We trained CNN models to predict neuronal subtype-specific activity of OCRs and used the models to predict whether addiction-associated genetic variants in risk loci impact putative CRE function. Our findings link the genetic heritability of addiction-associated behaviors to the OCR profiles of neuronal subtypes and brain regions and present specific hypotheses describing how genetic variants may impact gene regulation in addiction-associated behaviors. These analyses in conjunction suggest that genetic variation-associated nicotine, alcohol, and cannabis use behaviors may impact putative CREs in different combinations of excitatory (EXC), D1, and D2 neuronal subtypes. These findings provide a foundation for future investigations into the cell type-specific genetic mechanisms underlying addiction-related traits. More broadly, our cross-species integrative computational framework leverages high-resolution cell type-targeted epigenomics in model organisms to interpret the genetic risk variants of complex traits in humans.

**Figure 11.**
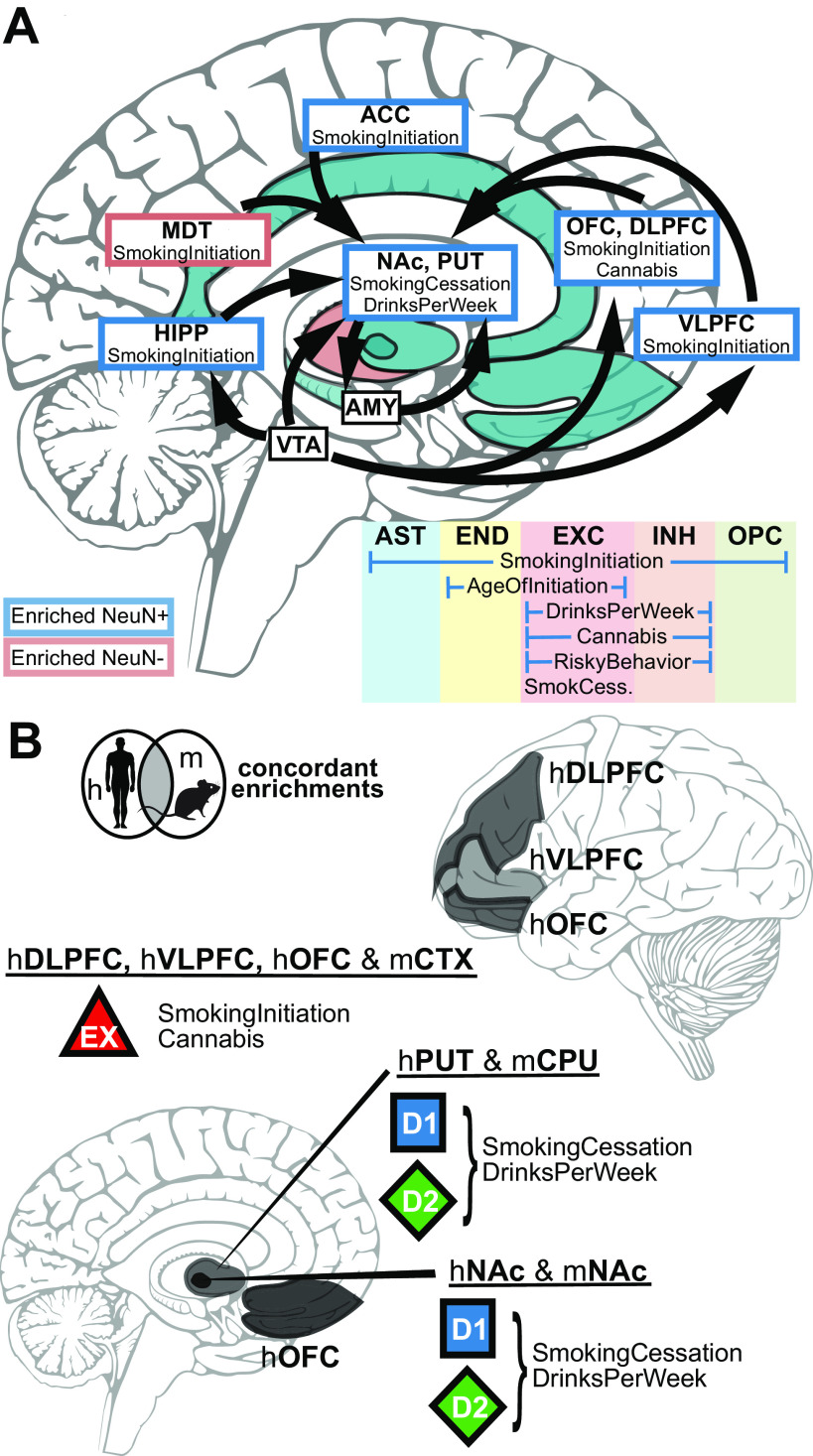
Summary of LDSC GWAS enrichments in human and mouse-human orthologous bulk tissue and cell type open chromatin. ***A***, Schematic of human NeuN-labeled bulk tissue and occipital cortex cell types from Figure 2*C*, for which addiction-associated genetic variants were significantly enriched (FDR < 0.05) in OCRs. Brain regions are labeled by the cell type that enriched (blue box/shading represents NeuN^+^; red box/shading represents NeuN^–^) spatially along with the trait(s) for which OCRs were found significantly enriched with risk variants. Occipital cortex cell types from Figure 5*C* are listed along with the trait(s) for which OCRs were found significantly enriched with risk variants. ***B***, Schematic of addiction-associated genetic variants that share enrichments from human brain regions and neuronal subtypes in both human and mouse-human orthologous open chromatin. Brain graphic adapted from Fullard et al. (2018).

We initially found that addiction-associated genetic variants were enriched in human NeuN^+^ OCRs of the PFC and striatum, known areas involved in addiction and reward circuitry (Volkow et al., 2013; Koob and Volkow, 2016) (Fig. 11*A*). Genetic variants associated with SmokingInitiation and Cannabis, initiating behaviors of substance use, were enriched in NeuN^+^ OCRs of prefrontal areas, including DLPFC, VLPFC, and OFC (Fig. 2*A*). These OCRs were predicted to be active in cortical EXCs of these brain regions (Fig. 8*B*). Addiction-associated genetic variants that enrich in OCRs of cortical EXCs in these areas may reduce corticostriatal activation from PFC to inhibit behaviors predisposing the initiation of substance use (Koob and Volkow, 2010, 2016; Volkow et al., 2013; Volkow and Morales, 2015). These genetic variants may contribute to reduced prefrontal self-control reward, leading to behaviors observed in addiction, such as impulsivity, reduced satiety, and enhanced motivation to procure drugs (Volkow et al., 2013; Volkow and Morales, 2015). In addition, we found enrichment of striatal NeuN^+^ OCRs for genetic variants associated with SmokingCessation and DrinksPerWeek (Fig. 2*A*). In Figure 8*B*, we showed that these genetic variants are predicted to affect open chromatin in both D1 and D2 MSNs, which are coordinators of mesocorticostriatal dopamine systems (Koob and Volkow, 2010, 2016; Volkow et al., 2013). Genetic variants affecting open chromatin in these MSN subtypes may predispose individuals to increased alcohol use (DrinksPerWeek) or decreased nicotine use (SmokingCessation), perhaps driving the neuroplastic changes in D1 and D2 MSNs observed in human and rodent drug dependence studies (Volkow et al., 1996, 1997, 2003; G. J. Wang et al., 1997; Fehr et al., 2008; Cheng et al., 2017; Wilar et al., 2019). While drug addiction has been attributed to various areas of the reward circuit, our investigations into heritable genetic risk for addiction-associated traits unravel how regulatory DNA sequence variation in OCRs of projection neurons in implicated areas link genetic risk to neural circuits to behavior.

Since key component cell types of the reward circuit, such as D1 and D2 MSNs, have not been profiled for high-quality open chromatin measurements in a human reference genome to our best knowledge, we leveraged high-quality mouse cell type open chromatin measurements using a cross-species OCR mapping framework. We first conducted ATAC-seq of MSN and interneuron subtypes in mouse brain to identify neuronal subtype-specific OCRs. Then, we used a multiple genome sequence alignment framework to identify the orthologous regions of the human genome. By leveraging reference-genome free CRE ortholog mapping tools, we retained high-quality cell type-specific measurements within relevant brain regions of the reward circuit, enabling analysis of cell populations from brain regions where we lack primary human open chromatin profiles. Across these brain regions, we found remarkably concordant enrichments of cell type OCRs between mouse and human profiles as well as shared enrichments between traits (Fig. 11*B*). Genetic variants associated with both SmokingInitiation and Cannabis enriched in mouse-human orthologous OCRs of cortical EXC (Fig. 5*B*), concordant with enrichments in human cortical NeuN^+^ OCRs (Fig. 2*A*), which were predicted to include EXC neurons (Fig. 9*B*). Genetic variants from these two traits showed replicable enrichment in human EXC neuron OCRs of isocortex and occipital cortex (Fig. 2*B*,*C*), providing strong evidence that genetic variation in cortical EXC OCRs confers susceptibility to nicotine and cannabis use behaviors. The enrichments of genetic variants associated with Cannabis in isocortical IN OCRs (Fig. 2*B*) and mouse-human orthologous OCRs of cortical PV neurons (Fig. 5*B*) suggest that genetic variation in cortical PV neuron OCRs also confer susceptibility of cannabis use behavior. Within striatal regions, D1 and D2 MSN mouse-human orthologous OCRs enriched for genetic variants of all measured addiction-associated traits (Fig. 5*A*), with strongest concordance in human OCRs for genetic variants associated with SmokingCessation and DrinksPerWeek (Figs. 2*A* and 11*B*). The enrichments in conserved OCRs of MSN subtypes in the dorsal striatum and NAc unsurprisingly emphasize known roles of MSNs of both areas to drive and maintain addiction behaviors (Ferguson et al., 2011; Ji et al., 2017). Our validations of enrichments both at the tissue and cell type level across human and human-orthologous OCRs agree with LDSC regression GWAS enrichments of noncoding regions around differentially expressed genes in DLPFC and NAc measured from postmortem human subjects who were diagnosed with opioid use disorder versus neuropsychiatric controls (Seney et al., 2020). Because of the conservation of reward circuit between mouse and human, our approach is able to unravel the cell types in which genetic variation at the epigenome level predisposes addiction-related traits even from measurements in organisms that have not been exposed to addictive substances. Further, this level of OCR conservation is present at the level of excitatory cell types in cortical brain regions (Yao et al., 2021). This may explain why we found enriched cell types in occipital cortex (Fig. 2*C*), which is not well defined for its role in addiction-related traits.

In an orthogonal approach to mapping mouse-human orthologous OCRs, we devised and trained CNN models to classify the neuronal subtype membership of mouse and human NeuN^+^ OCRs to refine GWAS enrichments of bulk tissue to the major neuronal subtypes of cortex and striatum. This approach can provide further validation for enrichments of human and mouse-human orthologous OCRs in cell types and tissues. Refinement of NeuN^+^ OCRs revealed that addiction-associated traits enriched for two clusters of cell types and brain regions. The first group, which displays concordant cortical excitatory enrichments between human and mouse, consists of SmokingInitiation and Cannabis (Fig. 8*B*); and the second group, which displays concordant D1 and D2 MSN enrichments, consists of SmokingCessation and DrinksPerWeek. A drawback of assigning human NeuN^+^ OCR membership to individual cell types lies in the considerably low representation of interneurons in both cortical and striatal neuron populations, as low as 12% in neocortex (Beaulieu, 1993; Lefort et al., 2009) and ∼5% in striatum (Tepper and Koós, 2017; Krienen et al., 2020). NeuN^+^ open chromatin profiles alone do not always capture OCRs unique to rare interneurons, some of which had OCRs identified by human single-cell assays and mouse-human orthologs enriched for addiction GWAS variants (Fig. 5*B*,*C*). As a result, we did not train CNN models for PV, SST, or VIP interneurons. However, the striking enrichments of OCRs from certain interneuron populations for addiction GWAS variants begin to demonstrate these populations' roles in the addiction neural circuits (Bracci et al., 2002; Lansink et al., 2010; Wiltschko et al., 2010; Ribeiro et al., 2018; Jiang et al., 2019; J. H. Lee et al., 2020; Schall et al., 2020).

The overall concordance of enrichments across human and mouse-human orthologous OCRs suggests a conserved regulatory code between mouse and human CREs. Correspondence in the neural circuitry has been well appreciated between human studies and rodent models of addiction (Berke and Hyman, 2000; Koob and Volkow, 2016; Farrell et al., 2018), and our study further demonstrates that mouse-human conserved OCRs may explain considerable heritability of addiction-associated traits. This makes animal models suitable not only for studying the neural circuits of addiction but also cell type-specific gene-regulatory mechanisms of addiction.

We used several selection criteria along with CNN models to predict the functional impact of genetic variants associated with addiction-related traits (Figs. 7 and 11; Extended Data Fig. 7-1). The fine-mapping pipeline described effectively narrows down a set of 14,790 SNPs to a putatively functional set of 55 Tier A candidate causal SNPs that can be experimentally tested to determine which brain regions and neuronal subtypes they would have function in. The candidate functional SNPs that our models prioritize demonstrate how a candidate SNP within a locus, such as rs7604640 (Fig. 9*B*), might act in distinct neuronal subtypes and brain regions. Cell type and brain region specificity adds complexity to identifying how genetic variation may alter gene regulation to predispose an individual to addiction-associated traits. Our approach often reported 1-4 candidates per loci, even in stretches of SNPs in perfect LD, such as the *MAPT-CRHR1* locus (Fig. 7*D*). This reflects the idea that many SNPs in the same loci are significantly associated with addiction because of LD with only one or a few causal SNPs and are unlikely to influence addiction-associated genetic predisposition. We report that many candidate SNPs that also overlap mouse-human orthologs from the same predicted cell type raise the idea that altering the conserved regulatory DNA sequence may be a mechanism of cell type-specific gene regulatory tuning in a population or even across species (Gjoneska et al., 2015).

Our study depends solely on assays of open chromatin as a proxy for putative CREs. While our study utilizes well-characterized cell types from *cre*-driver lines against a C57BL/6J genetic background, we recognize the limitation of relying on one mouse strain in light of evidence that mouse genetic backgrounds have unique tissue-specific open chromatin (Halow et al., 2021). Epigenetic assays for chromatin conformation, histone modifications, and methylation would further inform how putative CREs regulate nearby gene expression. While eQTL studies do not control for inflated associations because of LD and report gene expression differences from bulk tissue, we do note that our approach prioritizes several SNPs known to be *cis-*eQTLs in relevant brain regions, which indirectly affirms our framework's ability to select SNPs with functional impacts on gene regulation. Although *cis*-EQTLs are often not cell type- or tissue-specific, our findings of risk loci in brain regions implicated in addiction-related traits reflect a strength of our approach in discerning brain-specific signal. In order to validate our predictions, it will be necessary to further investigate candidate genetic variants, such as rs7604640 (Fig. 9*B*), in future studies using a fluorescence reporter assay or ISH studies. These methods can measure regulatory activity differences between risk and nonrisk alleles to verify our predictions of SNP impact on putative CREs and indicate whether the reported differences in regulatory activity are cell type-specific. The candidate SNPs we identified provide possible mechanisms linking differences in genetic makeup with the genes, cell types, and brain regions that could influence addiction and substance use behaviors (Fig. 9).
