# Supplementary material for: Addiction-Associated Genetic Variants Implicate Brain Cell Type- and Region-Specific Cis-Regulatory Elements in Addiction Neurobiology
Source: J Neurosci. 2021 Oct 27;41(43):9008–30. doi: 10.1523/JNEUROSCI.2534-20.2021 (PMC8549541; doi:10.1523/JNEUROSCI.2534-20.2021)
Supplement: Extended Data Table 4-1 — cSNAIL sample description: genotype, number of replicates, sex, region and number of replicates per region, and cell type information for cSNAIL samples. Download Table 4-1, DOCX file. [file ns-JN-RM-2534-20-s02.docx]

**Figure 4-1. cSNAIL sample description**

**Genotype, number of replicates, sex, region and number of replicates per region, and cell type information for cSNAIL samples.**

| Genotype | Replicates | Sex  (Female /Male) | Region and Replicate per region | Cell type |
| --- | --- | --- | --- | --- |
| C57BL/6 WT | N=4 | 2 F, 2 M | CTX= 4, CPU/NAc = 4 | bulk |
| *Pvalb*-cre | N=5 | 3 F (CTX)  1 F, 1 M (CPU/NAc) | CTX= 3, CPU/NAc = 2 | PV |
| *Sst*-cre | N=2 | 1 F, 1 M | CTX= 2, CPU/NAc = 2 | SST |
| *Drd1*-cre | N=2 | 2 F | CPU=2, NAc=2 | D1 |
| *Adora2a*-cre | N=2 | 2 F | CPU=2, NAc=2 | D2 |
